# Pre-neoplastic epigenetic disruption of transcriptional enhancers in chronic inflammation

**DOI:** 10.18632/oncotarget.7513

**Published:** 2016-02-19

**Authors:** Aline C. Planello, Rajat Singhania, Ken J. Kron, Swneke D. Bailey, David Roulois, Mathieu Lupien, Sérgio R. Peres Line, Ana Paula de Souza, Daniel D. De Carvalho

**Affiliations:** ^1^ Princess Margaret Cancer Centre, University Health Network, Toronto, ON, Canada; ^2^ Department of Morphology, Piracicaba Dental School, University of Campinas, Piracicaba, SP, Brazil; ^3^ Department of Medical Biophysics, University of Toronto, Toronto, ON, Canada

**Keywords:** DNA methylation, enhancers, chronic periodontitis, oral cavity squamous cell carcinoma

## Abstract

Chronic periodontitis (CP) is a chronic inflammatory disease independently associated with higher incidence of oral cavity squamous cell carcinoma (OSCC). However, the molecular mechanism responsible for this increased incidence is unknown. Here we profiled the DNA methylome of CP patients and healthy controls and compared to a large set of OSCC samples from TCGA. We observed a significant overlap between the altered DNA methylation patterns in CP and in OSCC, suggesting an emergence of a pre-neoplastic epigenome in CP. Remarkably, the hypermethylated CpGs in CP were significantly enriched for enhancer elements. This aberrant enhancer methylation is functional and able to disrupt enhancer activity by preventing the binding of chromatin looping factors. This study provides new insights on the molecular mechanisms linking chronic inflammation and tumor predisposition, highlighting the role of epigenetic disruption of transcriptional enhancers.

## INTRODUCTION

The inflammatory response is an important mechanism for clearing tissue damage. However, uncontrolled inflammatory responses can lead to chronic inflammatory diseases, including chronic periodontitis (CP). CP is a highly prevalent chronic inflammatory disease, with a prevalence of 47% in adults aged 30 years and older in the USA [1] and believed to affect a large proportion of the worldwide population [2]. Epidemiological data have demonstrated that CP patients have a significantly higher incidence of oral cavity squamous cell carcinoma [3, 4]. Similarly, other chronic inflammatory diseases have been implicated in increasing the incidence of site-specific tumors, including: intestinal inflammation resulting in colon cancer [5] and liver inflammation increasing prevalence of hepatocellular carcinoma [6]. However, the molecular mechanisms that connect chronic inflammation with cancer are not completely understood. Delineating these mechanisms may have an important impact in cancer treatment and cancer chemoprevention [6].

Remarkably, chronic inflammation has been frequently associated with widespread changes in the DNA methylation profile [7-9]. However, the functional role of these aberrant DNA methylation profiles in chronic inflammatory diseases and cancer predisposition is not clear. For instance, a recurrent epimutation in chronic inflammation is frequently observed in the intragenic region of *SOCS1* (Suppressor of cytokine signaling 1). This small gene contains a CpG Island (CGI) spanning the promoter region and the majority of the gene body. This CGI is known to be hypermethylated in several types of chronic inflammatory diseases including chronic hepatitis [10, 11] and obesity [12]. Importantly, the *SOCS1* CGI is also frequently hypermethylated in various cancer types [13, 14], and forms part of the CpG island methylator phenotype (CIMP) panel in colon cancer [15]. The functional consequence of intragenic *SOCS1* hypermethylation is currently unclear and there is conflicting data on the correlation between *SOCS1* gene expression level and *SOCS1* CGI hypermethylation. A number of reports suggest a correlation with gene repression [13, 16] while others suggest no correlation [14, 17].

The functional role of DNA methylation heavily depends on its genomic context. DNA methylation at CGI transcription start sites (TSS) is frequently associated with stable, long-term gene repression [18], while the anti-correlation between DNA methylation and gene expression is less evident at non-CGI TSSs [18]. There is a positive correlation between gene body DNA methylation and gene expression [19]. At transcriptional enhancers, DNA methylation patterns are highly variable. Some data suggests that CpG-poor enhancers are only active in the absence of DNA methylation [20], whereas the role of DNA methylation at CpG-rich enhancers is currently not clear.

Here, we show for the first time that a pre-neoplastic DNA methylome emerges in chronic periodontitis (CP) patients and that this modified pattern of DNA methylation in CP strikingly resembles the DNA methylation patterns of oral cavity squamous cell carcinoma. Moreover, the pre-neoplastic DNA hypermethylation is preferentially localized to transcriptional enhancers and, as such, can functionally suppress enhancer activity, altering gene expression patterns.

## RESULTS AND DISCUSSION

**Figure 1:**
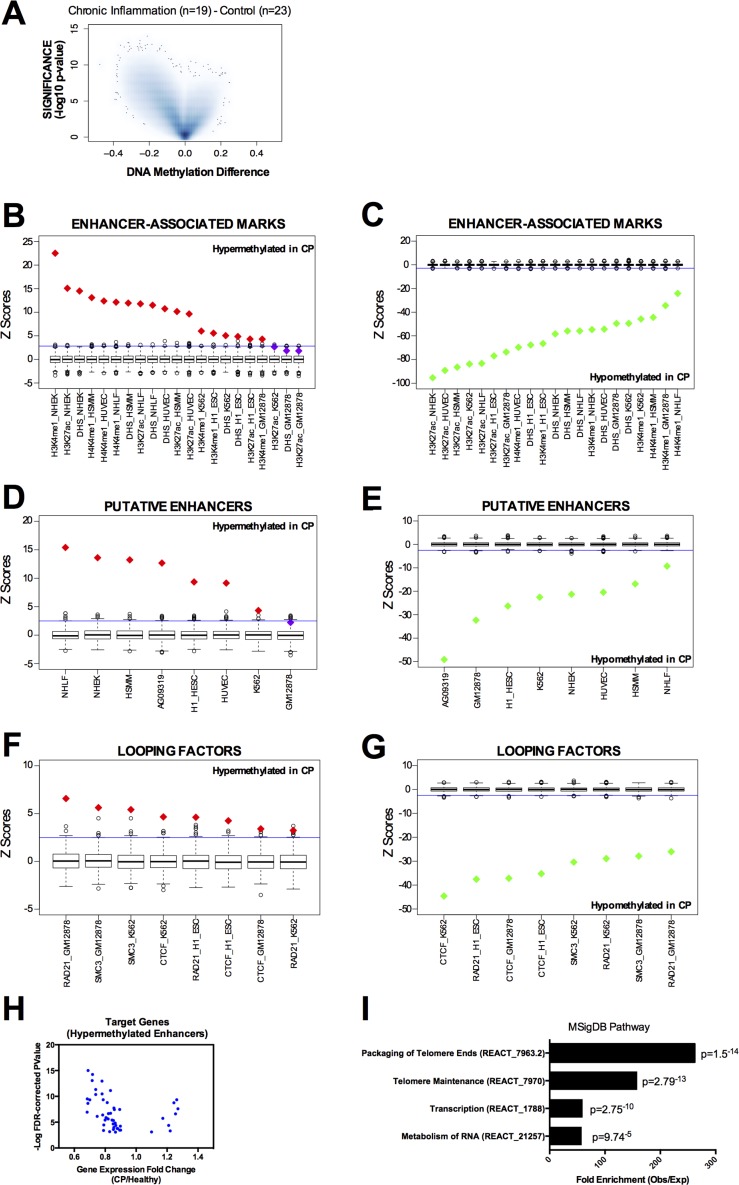
DNA methylation profile in chronic periodontitis highlights an aberrant DNA methylation at transcriptional enhancers **A.** Volcano Plot of all CpG loci analyzed. The beta value difference in DNA Methylation between chronic periodontitis (*n* = 19) and healthy controls (*n* = 23) is plotted on the x-axis, and the adjusted *p*-value of differences between the chronic periodontitis and healthy controls (−1* log10 scale) is plotted on the y-axis. **B.**-**C.** Overlap between differentially methylated CpG sites and enhancer-associated marks (H3K4me1, H3K27ac, and DHS) in tier 1 ENCODE cell lines (NHEK, HSMM, HUVEC, NHLF, K562, H1 and GM12878). Overlap was counted between each CpG and the peak for each mark defined by ENCODE. Box-plots represent 1,000 random permutations across the array of the same number of hypermethylated probes (left) or hypomethylated probes (right). Red diamonds represent the Z-score of significantly enriched marks and green diamonds represent Z-scores of significantly depleted marks. **D.**-**E.** Overlap between differentially methylated CpGs and putative enhancers (defined as DHS without the promoter-associated mark H3K4me3) in tier 1 ENCODE cell lines (NHEK, HSMM, HUVEC, NHLF, K562, H1 and GM12878) and in a human normal gingival fibroblast cell line (AG09319). **F.**-**G.** Overlap between differentially methylated CpGs and chromatin looping factors (RAD21, SMC3, and CTCF) in tier 1 ENCODE cell lines (NHEK, HSMM, HUVEC, NHLF, K562, H1 and GM12878). **H.** Volcano Plot for the putative target genes of ‘hypermethylated enhancers in CP’ identified by DHS correlation. Each dot represents one CpG. Expression data and FDR-corrected p-values were obtained from the GEO repository for chronic periodontitis and healthy tissues GSE10334 (247 CP samples from 183 diseased and 64 healthy sites). **I.** Gene set enrichment analysis using the putative target genes of ‘hypermethylated enhancers in CP’ against the MSigDB Pathway. Enrichment was performed using GREAT (Genomic Regions Enrichment of Annotations Tool). The coordinates of DHS correlations were used as input and the single nearest gene within 2.5 kb of the TSS was used to identify the promoter regions.

We analyzed the DNA methylation profile of gingival tissue from 42 age-matched individuals (Supplementary Table S1), 19 with chronic periodontitis diagnosis (CP group) and 23 with no clinical sign or symptoms of CP (healthy group) using the Infinium HumanMethylation450 BeadChip array - the same platform used by The Cancer Genoma Atlas (TCGA). Using a threshold of FDR corrected p-value lower than 0.05 and Beta value difference (CP minus control) higher than 0.15 (hypermethylated in CP) or lower than −0.15 (hypomethylated in CP), we identified 929 hypermethylated CpG sites and 40,535 hypomethylated CpG sites in CP tissue when compared to the healthy control group (Figure 1A). Hypermethylated CpGs were enriched for non-CGI regions; particularly open sea regions (defined as more than 4kb away from the closest CGI), compared to the expected array distribution (Supplementary Figure S1A). Furthermore, hypermethylated CpGs were enriched at intergenic and intronic regions, rather than promoter and exons (Supplementary Figure S1A), suggesting spurious hypermethylation in chronic inflammation may be interfering preferentially with distal cis-regulatory regions (enhancers) rather than proximal promoters. Previous studies have revealed that DNA methylation occurs more frequently within exons compared with introns in normal mammalian cells [21-23]. Our results suggest that during chronic inflammation, this normal DNA methylation pattern is disrupted (Supplementary Figure S1A).

Enhancer regions can be identified by the presence of histone modifications, including H3K4me1 and H3K27Ac [24], and DNase I hypersensitivity sites (DHS) [25]. In order to investigate whether the regions differentially methylated in CP were over-represented at distal regulatory regions, we overlapped them with enhancer marks in all seven ENCODE tier 1 cell lines (H1, NHEK, HSMM, HUVEC, NHLF, K562 and GM12878 cells). We first used the ENCODE tier 1 cells because of the wealth of data available (DHS, H3K27ac, H3K4me1, chromatin looping factors). To calculate the enrichment and significance, we performed 1,000 random permutations using the array distribution as the background (see Supplemental Methods). Indeed, hypermethylated CpGs were remarkably enriched for individual enhancer marks (Figure 1B) while hypomethylated CpGs were clearly depleted (Figure 1C). Next, we performed the same analysis using a more stringent definition of putative enhancers as DNase I hypersensitivity sites that do not overlap with the promoter mark H3K4me3. Again, we found that CpGs hypermethylated in CP are strikingly enriched at these putative enhancers in the majority of ENCODE cells assessed (Figure 1D), while hypomethylated CpGs were depleted (Figure 1E). Moreover, since transcriptional enhancer function is mediated by chromatin looping, we asked whether the differentially methylated CpGs in CP were preferentially localized at binding sites of chromatin looping factors. We investigated the cohesin complex subunits SMC3 and RAD21 [26], and CTCF, a protein that has been shown to co-operate with cohesin to promote the formation of chromatin loops [27]. Again, we found that CpGs hypermethylated in CP are enriched at chromatin looping binding sites in all the ENCODE cells available (Figure 1F), while hypomethylated CpGs were depleted (Figure 1G).

**Figure 2:**
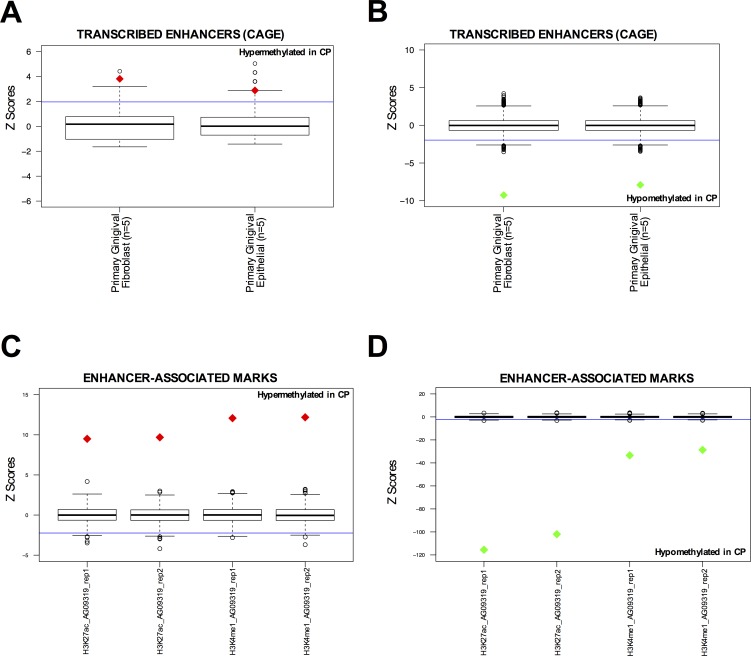
DNA hypermethylated sites in chronic periodontitis are enriched at normal enhancer elements in gingival tissues **A.**-**B.** Overlap between differentially methylated CpG sites and active enhancers (defined by bi-directonal CAGE-seq profile [28]) in primary gingival fibroblasts (*n* = 5) and primary epithelial (*n* = 5) tissues from FANTOM5 project. Overlap was counted between each differentially methylated CpG site and the called enhancer by FANTOM5 [28]. Box-plots represent 1,000 random permutations across the array of the same number of hypermethylated probes (left) or hypomethylated probes (right). Red diamonds represent the Z-score of significantly enriched marks and green diamonds represent Z-scores of significantly depleted marks. **C.**-**D.** Overlap between differentially methylated CpG sites and enhancer-associated marks (H3K27ac and H3K4me1) in normal gingival fibroblast (AG09319). ChIP-seqs were performed *in-house*, in duplicate. Overlap was counted between each differentially methylated CpG site and the peak for each mark called using MACS. Box-plots represent 1,000 random permutations across the array of the same number of hypermethylated probes (left) or hypomethylated probes (right). Red diamonds represent the Z-score of significantly enriched marks and green diamonds represent Z-scores of significantly depleted marks.

Additionally, since ENCODE tier 1 cell lines do not include gingival cell lines that more closely resemble the tissue of origin of CP, we also performed the same analysis on the available ENCODE data in two normal gingival fibroblast cell lines (AG09319 and HGF-1). We identified that hypermethylated CpGs in CP were enriched at DHS sites and CTCF binding sites in AG09319 and HGF-1, while the promoter mark H3K4me3 was not enriched (Figure 1D-1E and Supplementary Figure S1B-S1C). We performed the same permutation analysis on active enhancers in normal gingival fibroblasts (*n* = 5) and normal gingival epithelial (*n* = 5) tissues from the FANTOM5 project [28]. These active enhancers were called based on bi-directional transcription in CAGE-seq data [28]. Again, we observed that hypermethylated CpGs in CP were significantly enriched at active enhancers in normal gingival tissues (Figure 2A), while hypomethylated CpGs in CP were significantly depleted (Figure 2B). Finally, we performed *in-house* ChIP-seq (H3K27ac and H3K4me1), in duplicate, using normal gingival fibroblasts (AG09319 from Coriell Institute). Once more, we observed that hypermethylated CpGs in CP were significantly enriched at enhancer marks in normal gingival fibroblasts (Figure 2C), while hypomethylated CpGs in CP were significantly depleted (Figure 2D).

Together, these results highlight that the global DNA methylation pattern in chronic periodontitis is significantly altered when compared to normal tissue. Global DNA hypomethylation occurs outside of promoters, enhancers or CGIs; instead occurring mainly in intergenic regions or gene bodies. Furthermore, there is a focal DNA hypermethylation in CP tissue occurring preferentially at transcriptional enhancer regions.

To identify putative target genes of this set of hypermethylated enhancers in CP, we first identified each putative enhancer in the normal gingival fibroblast cell line (AG09319) by excluding the DHS that overlap with the promoter mark (H3K4me3). Then, we identified each potential enhancer in AG09319 that becomes hypermethylated in CP primary samples. We obtained a list of 127 hypermethylated putative enhancers in CP (Supplementary Table S2). Finally, to identify the target genes of these hypermethylated enhancers, we performed a pair-wise correlation analysis of DNase-seq profiles between each hypermethylated enhancer and surrounding DHS across a collection of over 100 human cell lines from 79 different cell types generated through the ENCODE project, as previously described [25, 29]. Previous studies have shown that this analysis can predict enhancer to promoter interactions with remarkable fidelity, when validated by 5C or ChIA-PET [25]. Restricting our analysis to a window of ±500kb surrounding each hypermethylated enhancer and using r^2^≥0.7 as a threshold for the pair-wise correlation, as previously described [25], we identified 60 putative target gene promoters (± 2.5KB around the TSS) (Supplementary Table S3). These genes have putative active enhancers in normal gingival fibroblast (AG09319) and DNA hypermethylated enhancers in CP. Indeed, gene expression meta-analysis of publically available cDNA microarray data [30] (GSE10334) of 247 samples (from 183 CP and 64 healthy sites) reveals that most of these genes are significantly down regulated in CP compared to normal gingival tissue (Figure 1H), suggesting that DNA hypermethylation can directly disrupt the transcriptional enhancer activity of these regulatory elements. These target genes are highly enriched at MSigDB for packaging and maintenance of telomeres, transcription and RNA metabolism (Figure 1I). Interestingly, in chronic hepatitis, another chronic inflammatory disease linked to higher cancer predisposition, telomere shortening during chronic inflammation is suggested to play a role in the progression to neoplasia [31].

**Figure 3:**
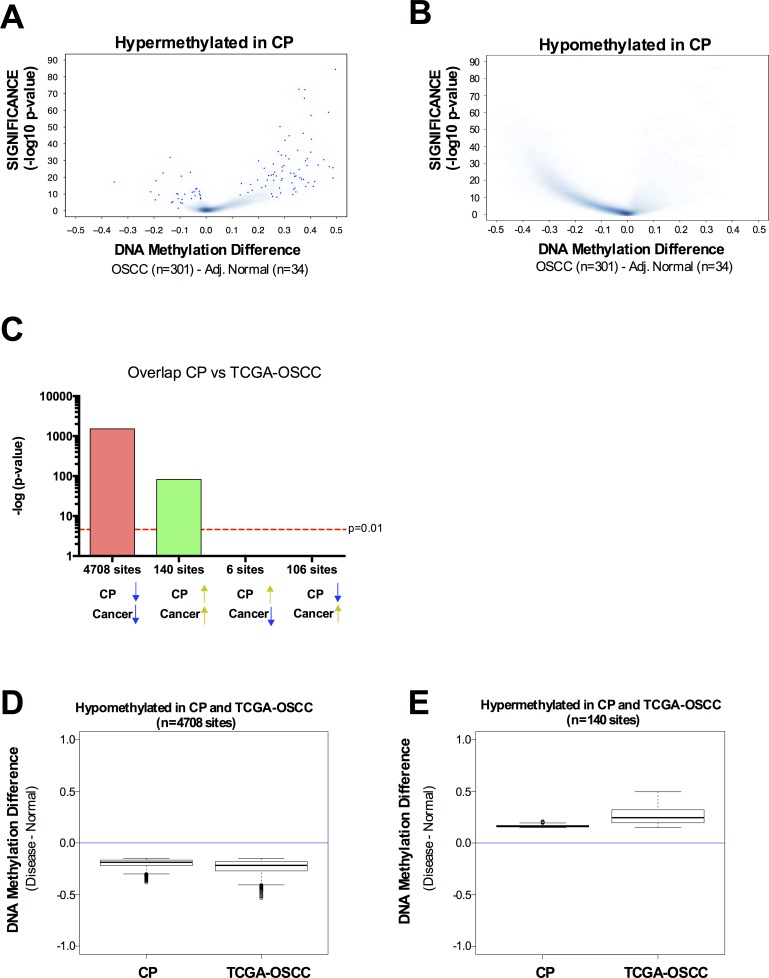
DNA methylation profile in chronic periodontitis highlights a pre-neoplastic DNA methylome **A.**-**B.** Volcano Plots of hypermethylated (A) or hypomethylated (B) CpG loci identified in CP *versus* normal. The beta value difference in DNA Methylation between Oral cavity carcinoma samples (*n* = 301) and adjacent normal tissue (*n* = 34) is plotted on the x-axis, and the adjusted p-value of differences between the chronic periodontitis and Healthy controls (−1* log10 scale) is plotted on the y-axis. **C.** Statistical comparison of the overlap and directionality of DNA methylation changes between CP *versus* Normal and OSCC *versus* Normal. Vertical bar represents the significance of the overlap between probe sets which was calculated using the hypergeometric test. Probes hypomethylated in CP significantly overlapped with probes hypomethylated in OSCC but do not significantly overlapped with probes hypermethylated in OSCC. Similarly, probes hypermethylated in CP significantly overlapped with probes hypermethylated in OSCC but do not significantly overlapped with probes hypomethylated in OSCC. We used the same parameters (*P*_adj_ < 0.05; Delta Beta > |0.15|) to identify CpG sites as differentially methylated for tumor samples *versus* adjacent normal, as we used for CP *versus* healthy controls. **D.**-**E.** Box-plots showing the magnitude of the differential methylation between CP *versus* healthy controls and tumor samples *versus* adjacent normal for the probes identified as hypomethylated in CP and in TCGA-OSCC **D.** and for the probes identified as hypermethylated in CP and in TCGA-OSCC **E.**

Since CP patients have a significantly higher incidence of oral cavity squamous cell carcinoma [3, 4], we sought to investigate whether the altered DNA methylation pattern observed in CP was also present in OSCC. We retrieved the DNA methylation data available from TCGA using the same platform (HumanMethylation450 BeadChip array) for 301 oral cavity squamous cell carcinoma samples and 34 adjacent normal samples. In general, hypermethylated CpG sites in CP were also hypermethylated in OSCC (Figure 3A) and hypomethylated CpG sites in CP were also hypomethylated in OSCC (Figure 3B). Indeed, there is a significant overlap between hypermethylated CpG sites in CP and hypermethylated CpG sites in OSCC (Figure 3C). The same is true for hypomethylated CpG sites in CP and hypomethylated CpG sites in OSCC (Figure 3C). Of the CpG sites identified as hypermethylated in CP and with available data at TCGA (*N* = 665), 21% were also hypermethylated in oral cavity carcinomas (hypergeometric probability = 1.498152^−36^) (Figure 3C). Less than 1% of the hypermethylated CpG sites in CP were hypomethylated in oral cavity carcinomas (Figure 3C). Conversely, of the CpG sites hypomethylated in CP with available data at TCGA (*N* = 26,396), 18% were also hypomethylated in oral cavity carcinomas (hypergeometric probability = 7.9^−197^) (Figure 3C). Again, less than 1% of the hypomethylated CpG sites in CP were hypermethylated in oral cavity carcinomas (Figure 3C). This data shows that CpG sites with differential methylation between CP *versus* normal and CpG sites with differential methylation between OSCC *versus* normal have an overlap significantly higher than expected by chance, and the differential methylation has the same directionality in CP and OSCC. Interestingly, although the directionality of the methylation change between CP *versus* normal and OSCC *versus* normal is the same, the magnitude of this change is higher in OSCC (Figure 3D-3E). Taken together, our data highlights a pre-neoplastic DNA methylome in chronic periodontitis patients, with a preferential hypermethylation of transcriptional enhancers.

**Figure 4:**
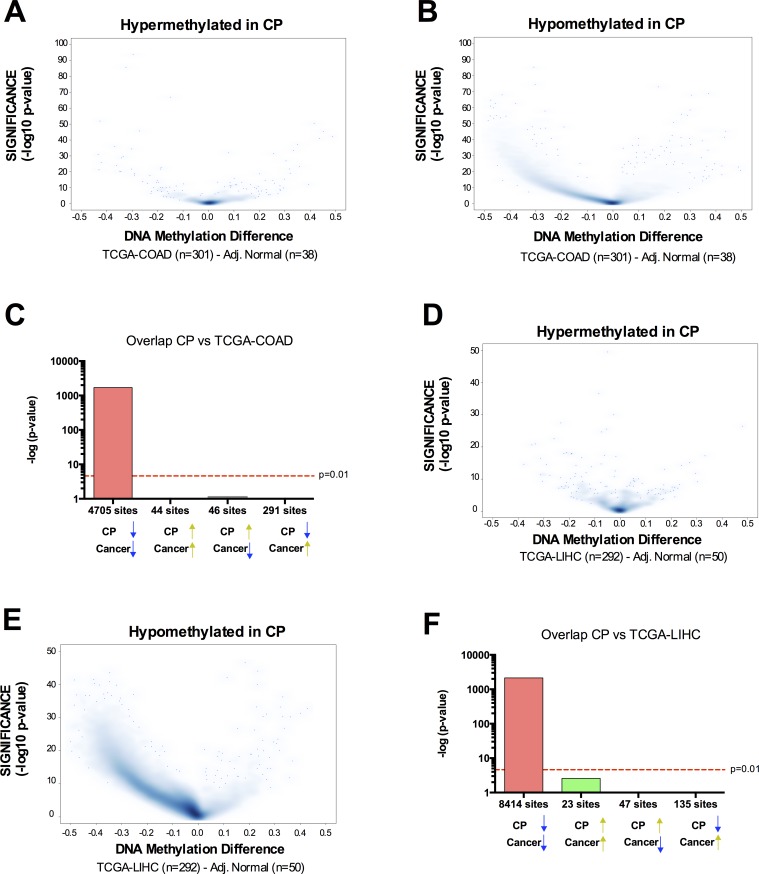
Overlap between chronic inflammation DNA hypermethylation and cancer hypermethylation is tissue type-specific, while overlap between chronic inflammation DNA hypomethylation and cancer hypomethylation is a general phenomenon **A.**-**B.** Volcano Plots of hypermethylated **A.** or hypomethylated **B.** CpG loci identified in CP *versus* normal. The beta value difference in DNA Methylation between Colorectal Adenocarcinoma samples (*n* = 301) and adjacent normal tissue (*n* = 38) is plotted on the x-axis, and the adjusted p-value of differences between the chronic periodontitis and healthy controls (−1* log10 scale) is plotted on the y-axis. **C.** Statistical comparison of the overlap and directionality of DNA methylation changes between CP *versus* normal and COAD *versus* Normal. Vertical bar represents the significance of the overlap between probe sets calculated using the hypergeometric test. Probes hypomethylated in CP significantly overlapped with probes hypomethylated in COAD but did not significantly overlap with probes hypermethylated in OSCC. On the contrary, probes hypermethylated in CP did not overlap with probes hypermethylated in COAD. We used the same parameters (*P*_adj_ < 0.05; Delta Beta > |0.15|) to identify CpG sites as differentially methylated for tumor samples *versus* adjacent normal, as we used for CP *versus* healthy controls. **D.-F.** Similar results were obtained for the overlap between CP and Liver Hepatocellular carcinoma (LIHC).

The implication of these results is that the DNA methylation pattern links CP to OSCC. To test whether this could also be a more general relationship between inflammation-related DNA methylation and cancer DNA methylation, we retrieved the DNA methylation data available from TCGA for 301 colorectal adenocarcinoma (COAD) samples and 38 adjacent normal samples; and 292 Liver Hepatocellular Carcinoma (LIHC) and 50 adjacent normal. We chose these two cancer types because they are known to have increased incidence following chronic inflammation: intestinal inflammation resulting in colon cancer [5] and liver inflammation increasing prevalence of hepatocellular carcinoma [6]. In contrast to OSCC, hypermethylated CpG sites in CP were not preferentially hypermethylated in COAD or LIHC (Figure 4), suggesting that the pre-neoplastic DNA hypermethylation identified in CP is site-specific and directly related to oral cancer but not other cancer types with a strong inflammatory component. However, hypomethylated CpG sites in CP were also significantly hypomethylated in COAD and LIHC (Figure 4), suggesting that the global hypomethylation observed in chronic inflammation is generally linked to the global hypomethylation observed in other cancer types.

**Figure 5:**
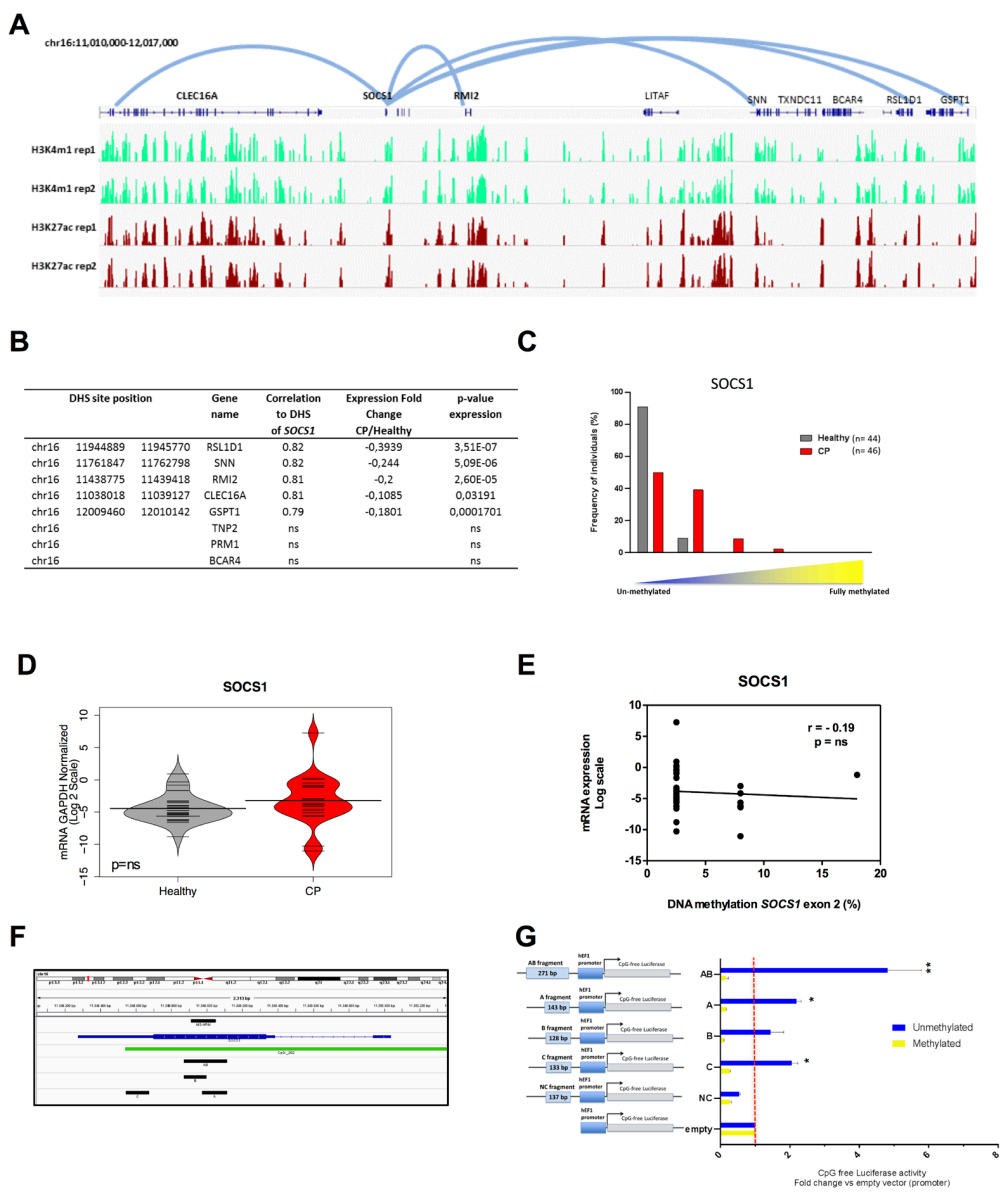
*SOCS1* acts as an enhancer element in normal gingival tissue and is hypermethylated in chronic periodontitis **A.** ChIP-seq profile for enhancer-associated marks (H3K27ac and H3K4me1) in normal gingival fibroblasts (AG09319) performed in duplicate highlights a strong enrichment of enhancer marks at SOCS1. The five predicted loops for the *SOCS1* enhancer are represented by the light blue lines (*CLEC16*A, *RSL1D1*, *RMI2*, *SNN* and *GSTP*1). **B.** Table showing DHS chromosome positions, gene names, correlation values, gene expression fold changes in chronic periodontitis relative to healthy tissue, and p-values of gene expression for the five predicted target genes plus three control genes within the same genomic window (TNP2, PRM1, and BCAR4). RNA expression data was obtained from GSE10334 for chronic periodontitis and healthy tissues (183 diseased and 64 healthy). **C.** DNA methylation of the exon 2 *SOCS1* region (chr16:11,348,911-11,349,051) in chronic periodontitis *n* = 46 (red) and healthy tissue *n* = 44 (gray) using MS-HRM. Results were categorized in groups by DNA methylation level, (0-5, 6-10, 11-25, and 26-50% methylation) and graphed based on percentage of individuals in each category and analyzed using Fisher exact test. *P* < 0.001. **D.** qPCR expression level of *SOCS1* gene. Data was transformed and normalized to GAPDH expression. Values are shown in log2 scale and groups were compared using Mann Whitney U test: *n* = 20 (CP), *n* = 25 (healthy). ns: non-significant. **E.** Correlation between *SOCS1* exon 2 methylation and gene expression. Methylation values are the mean of each MS-HRM group (e.g. 0-5% = 2.5%). **F.** Blue: *SOCS1* gene, green: CpG island, black: fragments used for the gene reporter constructs: A (chr16:11348973-11349115), B (chr16:11348872-11348999), C (chr16:11348544-11348676) and AB (chr16:11348872-11349115). **G.** Unmethylated and methylated CpG free-promoter-Lucia (Renilla) plasmids harboring *SOCS1* fragments in place of an enhancer were used. Negative control (NC): a genomic region without enhancer marks (chr15:67610118-67610254) (Supplementary Figure S2C). Relative luciferase units were normalized to firefly signal. The luciferase expression level for each fragment is relative to the empty vector. Replicates were 10, 50 and 100ng of the test plasmid and the luciferase levels were normalized to the transfected plasmid amount. All constructs were compared using T test with pooled SD and bonferroni corrected; (*) *p* ≤ 0.05; (**) *p* ≤ 0.01.

In order to validate our genome-wide data in a larger cohort of patients, we analyzed the DNA methylation profile of the *SOCS1* CGI in 90 age-matched individuals: 46 individuals with CP (CP group) and 44 healthy participants (healthy group) (Supplementary Table S1), via Methylation Sensitive High Resolution Melting (MS-HRM) [32] (Supplementary Figure S2A). This region was chosen due to its recurrent epimutation found in several chronic inflammatory diseases and cancer [10-15, 33] and because it falls within a region enriched for enhancer marks in normal gingival fibroblasts (Figure 5A). As with a number of other chronic inflammatory diseases, we observed that the intragenic *SOCS1* CGI was hypermethylated in CP tissue more frequently than when compared to normal tissue (Figure 5C). Indeed, exon 2 of the *SOCS1* CGI was completely unmethylated in 91% (41/44) of healthy tissue compared to just 50% (23/46) of chronically inflamed tissue. Of the 23 chronically inflamed samples that gained DNA methylation, 18 were 6-10% methylated, 4 were 11-25% methylated and 1 was 25-50% methylated. Conversely, just 4 healthy samples gained 6-10% DNA methylation (Figure 5C). Interestingly, the gain in DNA methylation observed in chronically inflamed tissues was not associated with a change in *SOCS1* gene expression itself (Figure 5D-5E), highlighting the potential of SOCS1 CGI to act as a distal cis-regulator rather than simply regulating its own expression.

Chronic inflammation is associated with the migration of inflammatory cells to the site of inflammation. Indeed, CP is histologically characterized by an infiltration of several inflammatory cell populations into the gingival epithelium and connective tissue; this includes T and B-lymphocytes, plasma cells, and macrophages among others [34-36]. The presence of these cells in CP tissue could therefore account for the hypermethylation of the intragenic *SOCS1* CGI observed in diseased tissue. To test this possibility, we re-analyzed a previously published genome-wide DNA methylation data-set [37] of purified immune-inflammatory cells, including: T and B cells; monocytes; natural killer (NK) cells; eosinophils and neutrophils. CpG sites located at the intragenic *SOCS1* CGI region were completely unmethylated in all types of immune-inflammatory cells evaluated (Supplementary Figure S2B, red box). This data suggests that the gain in DNA methylation seen in CP samples is probably not a consequence of immune cells infiltration. In fact, since CP gingival tissue has an increased number of immune-inflammatory cells[34-36], and these cells seems to be unmethylated at this region, the observed hypermethylation at the *SOCS1* CGI (Figure 5C) is likely to be underestimated.

To identify putative target genes of the enhancer located at *SOCS1* CGI, we performed a pair-wise correlation analysis of DNase-seq profiles between the *SOCS1* CGI and surrounding DHS across a collection of over 100 human cell lines from 79 different cell types generated through the ENCODE project. Restricting our analysis to the window of ±500kb surrounding the intragenic *SOCS1* CGI (chr16:11,348,911-11,349,051) identified DHS within the promoters of *RSL1D1 (CSIG), SNN, RMI2, CLEC16A*, and *GSPT1* genes as putative targets of the *SOCS1* CGI regulatory region (r^2^≥0.7) (Figure 5A). Gene expression meta-analysis of publically available cDNA microarray data [30] (GSE10334) of 247 samples (from 183 CP and 64 healthy sites) reveals that each of these genes was down-regulated in chronic periodontitis compared to healthy sites (Figure 5B). These genes are related to DNA repair [38], cell proliferation [39], apoptosis [40] and immune inflammatory regulation [41]. As a control, we randomly selected 3 genes out of the 18 genes located within the same window of ±500kb surrounding the *SOCS1* CGI regulatory region that do not present DHS correlation to this region (and therefore are not likely to have a promoter-enhancer interaction with *SOCS1* CGI): *TNP2, PRM1* and *BCAR4*. None of these genes were statistically significantly down-regulated in chronic periodontitis tissues (Figure 5B).

Next, we sought to investigate whether the observed enhancer DNA hypermethylation was functional and able to disrupt the enhancer function. We performed a luciferase assay using a reporter plasmid (human EF-1α promoter) completely devoid of CpG dinucleotides with a cloning site in place of the enhancer (Invivogen). Four fragments were cloned: two regions were located in or upstream of exon 2 of *SOCS1* CGI (fragments A and B); one fragment was downstream of the hypermethylated region but still contained in exon 2 CGI (fragment C), and a final fragment that contained both fragments A and B (fragment AB) (Figure 5F). A fragment that lies in a chromatin region devoid of enhancer marks in multiple cell types was cloned as a negative control (fragment NC) (Supplementary Figure S2C). The constructs were transfected into HEK293 cells and the luciferase activity was measured 24 hours after transfection. All results were normalized relative to the empty vector (promoter-only, without enhancer).

As shown in Figure 5G, all *SOCS1* fragments were able to significantly increase the activity of the EF-1α promoter, with fragment AB showing the strongest enhancer activity. Interestingly, we observed enhancer activity for fragment C. This indicates that the regulatory region of *SOCS1* can be extended further into exon 2. These results indicate a definitive enhancer activity for exon 2 of the *SOCS1* gene. Furthermore, when we performed *in vitro* DNA methylation of the same constructs, we were able to abolish the enhancer activity of fragments A, B, AB and C, without any effect on the negative control or the promoter-only empty vector (Figure 5G). Since the plasmid used for this study was completely devoid of CpG dinucleotides, the DNA methylation only occurred on the inserted enhancer fragments, confirming that DNA methylation is able to functionally suppress enhancer activity.

**Figure 6:**
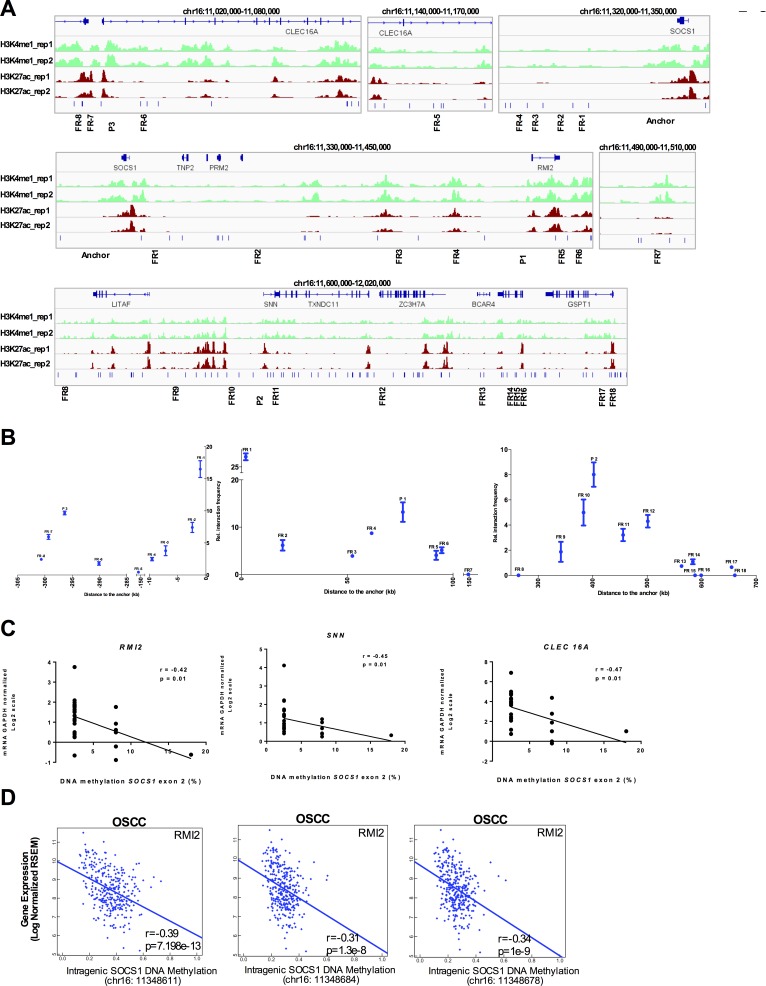
*SOCS1* enhancer physically loops to its target genes in normal gingival tissue and is disrupted in chronic periodontitis and OSCC **A.** Schematic representation of the 3C assays. The enhancer element located at SOCS1 was used as anchor (top panel). Each predicted target promoter was represented as ‘P’ (P1: RMI2; P2: SNN; P3: CLEC16A). Several Flanking Regions (FR) between the promoters and enhancers were used (FR-8 to FR18). **B.** 3C qPCR of long distance interactions assay on Gingival Fibroblasts (AG09319) using SYBR green. The relative interaction frequency of each ligation product to the anchor region (SOCS1 enhancer) has been plotted. Three independent 3C-qPCR experiments were performed. Error bars represent standard error of the mean. **C.** Spearman correlation between *SOCS1* gene body DNA methylation and the expression level (qPCR) of the validated target genes. **D.** Correlation of intragenic *SOCS1* CGI methylation and expression level of target gene (*RMI2*) in 301 oral cavity squamous cell carcinoma samples from TCGA. The DNA methylation data was obtained using the HumanMethylation450 platform (probes cg03014241, cg04004558, and cg10784813) and plotted as beta values. The gene expression data was obtained by RNA-seq and plotted as log2-transformed normalized count.

Next, we used the Chromatin Conformation Capture (3C) assay to physically validate the enhancer/promoter interactions. Using normal gingival fibroblasts (AG09319), we were able to detect a 76kb long distance interaction between intragenic *SOCS1* CGI (enhancer) and RMI2 promoter, a 308kb long distance interaction between intragenic *SOCS1* CGI (enhancer) and *CLEC16A* promoter and a 412kb long distance interaction between intragenic *SOCS1* CGI (enhancer) and *SNN* promoter (Figure 6A-6B), physically validating three out of five chromatin looping predictions. The chromatin interactions were further confirmed by sequencing the PCR products.

Indeed, we were able to identify a statistically significant negative correlation between the intragenic *SOCS1* CGI methylation and expression level of its target genes: *RMI2* (r = −0.42), *SNN* (r = −0.45), and *CLEC16A* (r = −0.47) in a subset of CP and healthy samples (n = 30) where we had DNA methylation (MS-HRM) and gene expression (real-time qPCR) data generated from the same tissue sample (Figure 6C). Moreover, the observed down regulation of the target genes (*RMI2, SNN*, and *CLEC16A*) in CP does not seem to be caused by changes in their own promoter DNA methylation (Supplementary Figure S2D). This data highlights the ability of pre-neoplastic enhancer methylation to alter the gene expression profile of the affected tissue. Interestingly, the negative correlation of *SOCS1* CGI methylation and expression of its target genes still holds true for *RMI2* (r = −0.39) in a larger set of 301 oral cavity squamous cell carcinoma patients from TCGA (Figure 6D).

To further validate our analysis, we chose three other predicted enhancers, based on the enhancer marks in normal gingival fibroblasts (AG09319) (Supplementary Figure S3A). These three enhancers were found to be hypermethylated in CP (Figure 1A). Again, we were able to validate the enhancer/promoter looping by 3C in AG09319 cells (Supplementary Figure S3A-S3B and Figure S4).

**Figure 7:**
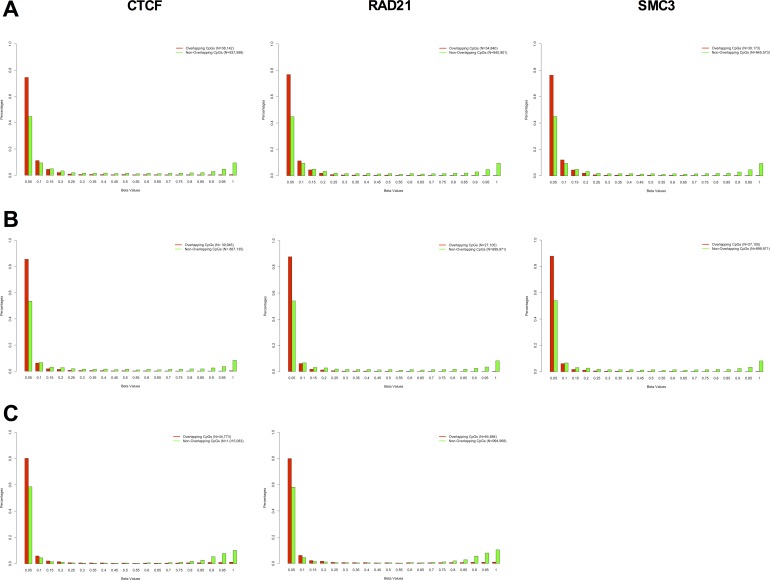
DNA methylation and chromatin looping factor binding is mutually exclusive DNA methylation profile of CpG sites overlapping with CTCF (left), RAD21 (middle) and SMC3 (right) binding sites (red bars), and DNA methylation profile of CpG sites not overlapping with CTCF (left), RAD21 (middle) and SMC3 (right) binding sites (green bars) in K562 **A.**, GM12878 **B.**, and H1-hESC **C.** The DNA methylation profile was obtained by RRBS (Reduced Representation Bisulfite Sequencing) from the ENCODE database and the looping factor binding sites were obtained by ChIP-seq from the same database using ENCODE called peaks. We evaluated the methylation sites of 975,740 CpGs in K562, 927,076 CpGs in GM12878 and 1,059,852 CpGs in H1 ESC with at least 5X coverage on the RRBS assay. There was a significant shift in the DNA methylation profile of overlapping *versus* non-overlapping CpGs in all eight panels (Kolmogorov-Smirnov test of Frequency distribution data, *P*-value < 1.326^−6^).

In order to establish a more general relationship between DNA methylation and long-range promoter/enhancer interactions, we analyzed eight ChIP-seq data-sets from ENCODE covering three different cell types (K562, GM12878 and H1 ESC) and three different chromatin looping factors (SMC3, RAD21, and CTCF) and 3 RRBS (reduced representation bisulfite sequencing) DNA methylation data-sets covering the same cell types. SMC3 and RAD21 are subunits of the cohesin complex and are necessary for physically and functionally connecting enhancers to the core promoters of active genes [26]. CTCF is a protein that has been shown to co-operate with cohesin to promote the formation of chromatin loops [27]. We were able to identify the DNA methylation level of approximately 1 million individual CpGs in each cell type with at least 5X coverage per CpG (975,740 CpGs in K562; 927,076 CpGs in GM12878; and 1,059,852 CpGs in H1 ESC). We measured the DNA methylation as a beta value, where zero corresponds to no methylation and 1 to fully methylated. We observed a significant mutual exclusivity between DNA methylation and looping factor occupancy, with the majority of CpGs within a looping factor binding-site being fully unmethylated (Figure 7). This data suggests that a potential mechanism for DNA methylation-mediated disruption of transcriptional enhancer activity might be by blocking or displacing the binding of chromatin looping factors.

Conversely, when we measured DNA methylation levels within peaks of enhancer-associated marks (H3K27ac, H3K4me1, DHS), we observed that the majority of the CpGs are unmethylated (Supplementary Figure S5).

Taken together, we have demonstrated that chronically inflamed tissues have a pre-neoplastic epigenome characterized by global hypomethylation and focal hypermethylation of enhancers. This enhancer hypermethylation is functional and can repress the transcriptional enhancer activity, ultimately altering the gene expression profile. Furthermore, since chronic inflammatory diseases have been implicated in the higher incidence of site-specific tumors [3, 5, 6] and DNA methylation of transcriptional enhancers has also been recently implicated in cancer predisposition [42-45], our work suggests that pre-neoplastic hypermethylation of transcriptional enhancers in chronic inflammatory diseases may play an important role in tumor development and may be a good target for cancer chemoprevention.

## MATERIALS AND METHODS

### Study approval and samples collection

The collection and analysis of chronic periodontitis cases and biopsies was carried out in accordance with protocols approved by Institutional Review Board at FOP/UNICAMP. Informed consent was obtained from all individuals at the time of sample collection. See Supplemental Methods for inclusion/exclusion criteria and collection protocols.

### DNA methylation assay

Genomic DNA (1 μg each) from chronic periodontitis tissue and healthy tissue were bisulfite converted and processed for the Illumina Infinium DNA methylation platform (HumanMethylation450 BeadChip) as previously described [46]. The Infinium methylation assays were performed by the Ontario Cancer Institute Genomics Centre (OCIGC) and by the USC epigenome center in accordance with the manufacturer's guidelines. The data was deposited on GEO (GSE59962). DNA methylation data from oral cavity squamous cell carcinoma and adjacent normal was obtained from TCGA (http://cancergenome.nih.gov/). See Supplemental Methods for detailed description of data analysis.

### Methylation sensitive high resolution melting (MS-HRM)

Real-time PCR followed by HRM was completed using a Light Cycler 480 II (Roche) (See Supplemental Methods).

### Bioinformatics

Pearson correlation coefficient (r) was calculated between DNaseI hypersensitivity signal intensities from all ENCODE cell lines with available DNaseI-Seq data (See Supplemental Methods).

### Chromatin immunoprecipitaiton and ChIP-seq library preparation

ChIP assays were performed by crosslinking ∼5 million cells (Gingival fibroblasts; AG09319, Corriell Institute). Four μg of antibody for H3K4me1 (Abcam ab8895 lot GR61294-1) and H3K27ac (Abcam ab4729 lot GR183919-2) were coupled to 10 μL of Dynabeads A and 10 μL of Dynabeads G (Invitrogen 10001D and 10004D, respectively) per ChIP (See Supplemental Methods).

### 3C-qPCR

Chromosome conformation capture (3C) was performed in three biological replicate in 7 million Gingival Fibroblasts (AG09319,Coriell Institute). The 3C library preparation followed three previously published reports [47-49]. 3C interaction products were detected by qPCR using SYBR green with candidate primer pairs (anchor and bait/controls) with the anchor primer placed in the fragment containing SOCS1 (See Supplemental Methods).

### *In vitro* analysis of SOCS1 enhancer and the influence of DNA methylation on its activity

Inserts were cloned into the CpG free-promoter-lucia plasmid (Invivogen) using InFusion HD Enzyme (Clontech). Fragments were treated (or not) with SssI DNA methyltransferase. Luminescence was measured using the Dual Luciferase Reporter Assay System (Promega, Madison) and the GloMax Multi+ Luminometer (Promega, Madison) (See Supplemental Methods).

## SUPPLEMENTARY MATERIAL FIGURES AND TABLES








