# Supplementary material for: Pre-neoplastic epigenetic disruption of transcriptional enhancers in chronic inflammation
Source: Oncotarget. 2016 Feb 19;7(13):15772–86. doi: 10.18632/oncotarget.7513 (PMC4941276; doi:10.18632/oncotarget.7513)
Supplement: Supplementary file 1 [file oncotarget-07-15772-s001.pdf]

Pre-neoplastic epigenetic disruption of transcriptional enhancers in chronic inflammation

Supplementary Material

Methods

Sample collection

Fresh gingival biopsies were obtained from 46 patients diagnosed with chronic periodontitis[1, 2] and 44 healthy controls exhibiting no signs of periodontal disease who had surgery for aesthetic reasons and/or 3rd molar excision. All individuals did not smoke, had no systemic disorder that could affect the periodontal condition, were not on antibiotics or anti-inflammatory medication (within the past six months), and were neither pregnant, lactating nor alcoholics. The samples were obtained from School of Dentistry of Piracicaba, University of Campinas (FOP/UNICAMP). Written informed consent was recorded from all individuals and the study was approved by the FOP/UNICAMP Ethics Committee.

In all cases gingival biopsies were obtained from a single tooth and were composed of junction epithelia and connective tissue.

All samples were collected immediately after surgery and stored in a nucleic acid conserver (RNA holder, Bioagency, São Paulo, SP, Brazil) and stored at -80°C, until the moment of nucleic acid extraction.

RNA and DNA extraction and bisulfite treatment

Total RNA was extracted and purified using the standard TRIZOL protocol (Invitrogen, Carlsband, CA, USA). Phenol/chloroform/ethanol protocol was completed following RNA purification allowing for DNA harvest. RNA and DNA quality was assessed on agarose gels.

DNA was bisulfite converted using the MethylSEQr Bisulfite Conversion kit (Life, Carlsbad, CA, USA) following the manufacturer’s protocol. Bisulphite converted DNA was utilized in MS-HRM.

Methylation Sensitive High Resolution Melting (MS-HRM)

Real-time PCR followed by HRM was carried out using a Light Cycler 480 II (Roche, Mannheim, Germany). Primer set design followed guidelines proposed by Wojdacz[3] and are shown in Supplementary Table S4. The reaction mixture consisted of 18ng bisulfite-converted DNA, 1x LightCycler®480 HRM Master Mix (Roche, Mannheim, Germany), 150nM of each primer, 3mM of $MgCl_2$ in a final volume of 20µl. The reaction conditions were: 1 cycle of 95°C for 10 minutes, 40 cycles at 95°C for 10 seconds, 55°C for 4 seconds and 72°C for 10 seconds. MS-HRM analysis was performed at the temperature ramping and fluorescence acquisition settings recommended by the manufacturer; 1 minute at 95°C, hold at 70°C for 1 minute (to allow re-annealing of all PCR product), acquisition step ramping from 70°C to 95°C, rising by 0.2°C/second with 25 acquisitions per °C.

To estimate the methylation level of each sample, converted fully methylated and fully unmethylated DNA (EpiTect PCR Control DNA Set from Qiagen) were used to prepare dilution series. The dilution series of relevant methylated DNA in a background of unmethylated DNA were prepared at concentrations of 5, 10, 25, 50, 75 and 100% methylation (providing a standard curve). PCR bias toward unmethylated DNA was reversed by following the guidelines published by Wojdacz. Standard curves and no template controls were included in each experimental run. As a negative control, genomic unconverted DNA from a pool of 3 healthy individuals was tested once with each primer pair. Initial assays were first run using 50% and 0% dilution controls until the proportionality of amplification between unmethylated and methylated was achieved. In order to compensate for varying starting fluorescence levels MS-HRM data was normalized (Light Cycler 480 II analysis software). The amplicon melting profile of each sample was compared to standard curves allowing each individual to be classified into a methylation category; 0-5, 6-10, 11-25, 26-50, 51-75 or 76-100%.

Expression Analyses

One microgram of total RNA was treated with DNase I RNase free (Life, Carlsband, CA, USA) and used for cDNA synthesis with First strand (Roche, Mannheim, Germany) following the manufacturer´s recommendations. Real Time qPCR of the

mRNA was completed using a Light Cycler 480 II (Roche, Mannheim, Germany). The reaction was carried out in a total volume of 20µl containing 1x LightCycler®480 Sybr Green (Roche, Mannheim, Germany), 250nM of each primer and 2µl of cDNA. PCR conditions were: 95°C 10 min, 40 cycles at 95°C for 10 seconds, appropriate temperature for each primer (Supplementary Table S5) for 10 seconds, 72°C for 10 seconds. Determination of relative gene expression level was performed using the cycle threshold (Ct) method in reference to GAPDH.

Bioinformatics

Chromatin Looping Analysis

Thurman *et al* observed that highly correlated DNaseI-Seq signal intensities ($r > 0.7$) calculated across a panel of 79 different cell types were enriched with chromatin interactions identified through 5C or ChIA-PET[4]. We applied this approach to predict chromatin interactions at the chr16:11,348,911-11,349,051 locus. We calculated the Pearson correlation coefficient (r) between DNaseI hypersensitivity signal intensities from all ENCODE cell-lines with available DNaseI-Seq data. We calculated the correlation in a cell type-specific manner, restricting the analysis to only DHS sites identified within AG09313, HEEpiC, Th1, Th2 and CD4+cells. In addition, we restricted our analysis to ±500kb surrounding the DHS anchor site that contained our region of interest. The DNaseI-Seq data was download directly from the ENCODE website (http://hgdownload.cse.ucsc.edu/goldenPath/hg19/encodeDCC/wgEncodeUwDnase/)[5].

Putative Enhancers

For Figures 1D and 1E, we calculate the putative enhancers by first downloading the DNaseH peak files for the 8 cell lines in consideration from ENCODE. We also download the peaks for each cell line for H3K4me3, and then use bedtools to filter out the DNaseH peaks which overlap H3K4me3 peaks. This yields our putative enhancers.

TCGA DNA Methylation Analysis

TCGA data was downloaded for head and neck squamous cell carcinoma (HNSC) from TCGA data-portal (https://tcga-data.nci.nih.gov/tcga/) in May 2014. Only samples from within the oral cavity were analyzed. Normalized DNA methylation data (Level 3) was obtained as a beta value for each sample. Level 3 TCGA data is background corrected using the 'noob' (Normal-exponential using out-of-band probes) method with dye-bias normalization. Gene Level normalized gene expression data from RNA-seq was obtained for each sample as a RSEM normalized count. All TCGA sample IDs used in this study are given in supplemental table S8. The filters applied on TCGA were "JHU-USC HumanMethylation450" for methylation, and "UNC IlluminaHiSeq_RNASeqV2" for gene expression. For probe selection, we analyzed all probes located within intragenic *SOCS1* CGI with the highest inter-patient variability (cg03014241, cg04004558, cg10784813) to calculate the correlation with gene expression of target genes.

Chronic Periodontitis DNA Methylation Analysis

A major goal of this study was to find CpGs differentially methylated in chronic inflammation as compared to normal samples. Raw DNA methylation data (Illumina HumanMethylation450) was analyzed from 19 Chronic Periodontitis (CP) and 23 control (healthy) samples. The arrays were performed in two different sites: 10 CP samples and 12 control samples were performed at the Ontario Cancer Institute Genomics Centre (OCIGC), and 9 CP and 11 control samples were performed at the USC (University of South California) epigenome center. All data was provided in IDAT files. Initially, these 4 sets of files were read one at a time by using the function read.450k() from the minfi package [6]. Next, the resulting 4 objects were subjected to background correction using the same method as Level 3 TCGA data (using the 'noob' (Normal-exponential using out-of-band probes) method with dye-bias normalization) as implemented in minfi. The resulting "MethylSet" objects were converted to the needed Beta values by the minfi function getBeta(). The Beta values can be directly obtained on GEO (GSE59962). In order to enable comparison of DNA methylation levels of the CpGs between CP and the control samples, we first had to correct for batch effects. This was done with the package sva using the

ComBat() function which uses an empirical Bayes method [7]. This function takes as input (i) the combination of the Beta values of each CpG from the CP samples processed at the two different sites (OCI & USC), and separately, (ii) the combination of the Beta values of each CpG from the OCI & USC control samples. The two resulting objects were then input together into another minfi function called dmpfinder() to find CpG positions having high Beta values in all CP samples compared to control, and vice-versa, using an F-test. Note that in this case, the dmpfinder() input parameter phenotype is not "continuous", but "categorical", because the phenotypes of interest are clearly defined as "CP" & "control". dmpfinder() yields the p-value and q-value (FDR corrected p-value) for each CpG comparison; in addition, we calculate, for each CpG, the difference (Delta) between the mean Beta value from the CP samples and control samples. In order to get our final list of 929 CpGs hypermethylated in CP, the criteria for Delta Beta value is > 0.15, and for q-value is < 0.05. Similarly, for the 40,535 CpGs hypomethylated in CP, the criteria for Delta Beta value is < -0.15, and for q-value is again < 0.05. The exactly same thresholds were used for calling hypermethylated and hypomethylated probes on the TCGA (OSCC, COAD, LIHC) analysis.

<u>ENCODE chromatin analysis</u>

ENCODE files from ENCODE tier 1 cell lines plus gingival cell lines (AG09319 and HGF-1) were downloaded to conduct the enhancer enrichment analyses. The chosen enhancer marks were DHS (DNase I Hyper Sensitive) sites, CTCF, H3K27ac and H3K4me1. A total of 39 BED files containing the peak called regions were collected from ENCODE database. We also included two BED files containing H3K4me3 peaks from AG09319 to exclude promoter regions, since there was no H3K27ac and H3K4me1 data available for this cell line.

Firstly, the occurrences of CpGs hypermethylated (and separately, hypomethylated) in CP falling within these BED files were calculated to yield the number of "biological intersections". Secondly, the "random intersections" were calculated by shuffling the corresponding hypermethylated (and hypomethylated) CpGs 1,000 times across the genome-wide 450K array of CpGs, and then intersecting the results of these shuffling with the BED files containing the peaks positions. Next, for each of the 39

cases, the number of biological and random intersections was compared to yield either enrichment or depletion of the biological intersection compared to the random. The cumulative probability distribution at the specified value (biological overlap) was calculated using the pnorm() function in R. The normality in the distribution of the number of random intersections was assessed using normal Q–Q plots.

Z-scores calculation

After obtaining the observed (“biological”) and expected (“random”) overlaps between CpGs of interest (e.g., hypermethylated in CP) and features of interest (e.g., enhancer marks, looping factors), we calculate the Z-score of the observed value taking into account the distribution of the expected values. We use 1000 permutations of the CpGs across the 450K array, and, after overlapping with each feature of interest, are able to get 1000 expected values following a normal distribution. The Z-score of each biological overlap is calculated by subtracting the mean of the expected overlaps from the observed value, and then dividing that number by the standard deviation of the expected overlaps. When plotting, we scale the expected values by subtracting mean and dividing by standard deviation, allowing us to put the biological overlap and random overlaps on the same scale.

To show which biological Z-scores are significant with respect to their random Z-scores, we calculate a cutoff Z-score which corresponds to a Bonferroni adjusted p-value of 0.05. For this, we first take into account the number of features. For example, in Fig 1D, we have 8 putative enhancers. A p-value is calculated for each enhancer with the R function pnorm() which measures the significance of each biological intersection with respect to the random intersections. Next, we calculated a Bonferroni adjusted p-value by simply multiplying the p-value with the number of p-values N, which in this case is 8. We want these corrected p-values to be less than our threshold of 0.05. Mathematically, we want $Pval * N < 0.05$; in other words, we want $Pval < 0.05/N$, to yield the uncorrected P-value for the threshold. Then, to calculate the cutoff Z-score, we use the R function qnorm() by providing this uncorrected P-value as the input probability parameter. The return value of qnorm is the number whose cumulative distribution matches the probability. This return value

is our cutoff Z-score. Note that because we scale the random intersections, the qnorm() function can use its default parameters of mean=0 and standard deviation=1.

Chromatin immunoprecipitaiton and ChIP-seq library preparation

ChIP assays were performed by crosslinking ~5 million cells in 1% formaldehyde (in PBS) at room temperature for 10 minutes. Crosslinked cells were then washed once with ice-cold PBS+BSA (5 mg/mL) and once with ice-cold PBS. Cells were then lysed in 320 µL lysis buffer (1% SDS, 10mM EDTA, 50 mM Tris-HCl pH8.1) and sonicated using a Bioruptor 300 on high setting for 25 cycles of 30 seconds on/30 seconds off. Insoluble cell material was cleared by centrifuging samples at 21,000 x g for 15 minutes at 4°C and removing the supernatant. 5 uL of each sample was removed and combined as an input and 1.6 mL of ChIP dilution buffer (1% Triton X-100, 2 mM EDTA, 150 mM NaCl, 20 mM Tris-HCl pH8.1) was then added to each sample. Four µg of antibody for H3K4me1 (Abcam ab8895 lot GR61294-1) and H3K27ac (Abcam ab4729 lot GR183919-2) were coupled to 10 µL of Dynabeads A and 10 µL of Dynabeads G (Invitrogen 10001D and 10004D, respectively) per ChIP. Antibodies and washed Dynabeads were incubated with rotation at 4°C in 300 µL of PBS+BSA for 6 hours. Coupled antibody/beads were then washed twice with PBS+BSA, resuspended in 110 µL of ChIP dilution buffer, and added to chromatin samples. Chromatin/antibody/bead mixtures were then incubated with rotation at 4°C overnight. The next day, samples were washed 3 times with cold RIPA-ChIP buffer (50 mM HEPES, 1 mM EDTA, 0.7% sodium deoxycholate, 1% NP-40, 0.5 M LiCl) with a 5 minute incubation and rotation for each wash. Immunoprecipitated chromatin was then washed twice with cold TE buffer before being resuspended in decrosslinking buffer (1% SDS, 0.1 M NaHCO3 and incubated at 65°C for ≥ 6 hours. Decrosslinked DNA was purified using a Qiagen Minelute kit (Qiagen Inc, Valencia, CA) according to the manufacturer’s recommended protocol.

Libraries for each ChIP were prepared using the Rubicon Thruplex FD kit (Rubicon Genomics, Ann Arbor, MI) according to the manufacturer’s recommended protocol. Following library amplification, samples were again purified using a Qiagen MinElute kit and eluted in 10 µL water. We then size selected libraries between 240-360 bp using the PerkinElmer LabChIP XT DNA 750 kit (PerkinElmer, Waltham, MA) according to the manufacturer’s recommended protocol. Samples were eluted in 20

µL elution buffer and submitted for 50 bp single-end sequencing at the Princess Margaret Genomics Centre using an Illumina HiSeq 2000. Five samples (two each of H3K4me1 and H3K27ac and one input sample) were sequenced in one lane, obtaining > 25 million reads for each.

ChIP-seq data analysis

The generated fastq files were aligned to the hg19 version of the human genome using Bowtie2 aligner with default parameters. Duplicate reads and reads with a mapq value <10 were removed using Samtools v0.1.19. H3K4me1 and H3K27ac peaks were then called using MACS v1.4 with default parameters set. Bedgraph files were generated using HOMER software v4.7.

3C-qPCR

Chromosome conformation capture (3C) was performed on Gingival Fibroblasts (AG09319,Coriell Institute) . The 3C library preparation followed two previously published reports[8, 9] with modifications suggested by Court et al 2011[10].

Briefly, 7 million Gingival Fibroblasts had the interacting chromatin segments cross-linked with 1% formaldehyde for 10 min at room temperature with rocking. The reaction was quenched with glycine. Following washing and incubation with permeabilization buffer, the nuclei of the cells were digested with Hind III by adding serially 150 units (U) of restriction enzyme for a total of 450 U. We started adding 150U of Hind III-HF and incubating at 37°C for 2h shaking at 900 rpm, then added gently for a second time more 150 U of Hind III and incubate at 37°C for 2h shaking at 900 rpm, then add again 150 U of HindIII for an overnight digestion at 37°C shaking at 900 rpm. To inactivate restriction enzyme, SDS (final 1.6%) was added and incubate for 30 min at 37°C. The cross-linked and digested DNA was ligated at low DNA concentration (T4 ligase 4000 units, 4h at 16 C and then 30 min at room temperature.) Cross-links were reversed by incubation at 65°C for 16 h in the presence of Proteinase K (40 µg/ml) followed by phenol/chloroform/ethanol DNA clean up.

3C interaction products were detected by qPCR using SYBR green with candidate primer pairs (anchor and bait/controls) (Supplemental Table S7). The reaction for detecting 3C interaction was performed using KAPA Sybr Fast qPCR Master Mix, 175 nM of the anchor forward primer and bait/controls reverse primers (final concentration) and 20 ng of 3C libraries (adjusted after quantification). Samples were tested in triplicate for amplification detection. The PCR conditions were 95 °C for 3 min followed by 40 cycles of 5 s at 95 °C, annealing and extension 30 s at 64 °C. The qPCR results from 3C-processed sample were normalized to serial dilutions (standard curve) of 3C-positive control template on each plate. The positive control was generated by synthesis of all possible PCR products using the available primers, followed by gel extraction and purification. PCR products were mixed in equimolar concentrations and digested with Hind III and purified by phenol/chloroform extraction and ethanol-acetate precipitation. The digested fragments underwent random ligation (T4 ligase) at high DNA concentration and purified with MiniElute PCR. To mimic 3C sample condition, the concentration of control template was adjusted by addition of genomic DNA that had undergone digestion and random ligation to the control template, increasing the complexity of the control. This way the PCR efficiency was not affected by the total amount of the DNA present (only the region of interest in the control template).

We used a published normalization method for data analysis[8]. The final value was calculated using value= 10 (Ct-b)/a (b: intercept and a: slope). These values were normalized to an internal control (GAPDH).

The interacting fragment of the anchor and bait was confirmed by Sanger sequencing.

In vitro **analysis of the** ***SOCS1*** **enhancer and the influence of DNA methylation on its activity**

<u>Insert amplification</u>

Genomic DNA was isolated from human venous blood using the QIAamp DNA Blood Kit (Qiagen, Hilden). The exon 2 region of *SOCS1* (chr16:11348911-11349051) was amplified using Kapa Hifi PCR kit (Kapa Biosystems, Massachusetts, USA) and 50ng of gDNA (Supplementary Table S6). Four fragments were cloned; two covering

the region of *SOCS1* found to be hypermethylated on the MS-HRM assay (A [chr16:11348973-11349115] and B [chr16:11348872-11348999]), one downstream fragment (in exon 2) at the end of the CGI (C [chr16:11348544-11348676]) and a final fragment at the junction of fragments A and B (AB [chr16:11348872-11349115]) (Figure 3C). A fragment which lies in a chromatin state devoid of enhancer marks was also cloned as a control (NC [chr15:67610118-67610254]). (Supplementary Figure S3A). PCR were performed as follows: 95°C 5 min; 98°C 20 sec, 60°C 15 sec, 70°C 30 sec (30 cycles). The fragments were gel purified using MiniElute Gel Kit (Qiagen).

Cloning of Luciferase expression vectors

BamHI and ScaI HF-linearized CpG free-promoter-Lucia (human EF-1α promoter) coelenterazine-utilizing luciferase (like Renilla) reporter plasmid (Invivogen) was used to clone the fragments with adapter sequences in place of the enhancer by recombination using InFusion HD Enzyme (Clontech Laboratories Inc., Mountain View, CA) at a ratio of 50ng vector: 50ng insert. Competent *E. coli* GT115 (Invivogen) were transformed with 2.5µL recombined plasmid and plated on LB-Agar Fast-Media Zeo Agar (Invivogen). A number of colonies were transferred to 3mL Fast-media Zeo TB (Invivogen) and plasmids isolated using the Pure link HiPure plasmid kit Miniprep (Life Technologies). After verifying the plasmids contained the fragments (restriction digestion of the plasmids with BamHI and ScaI), the transformed bacteria were transferred to 50ml Fast-media Zeo TB (Invivogen) and isolated/purified with Pure link HiPure plasmid kit Midiprep (Life Technologies).

In vitro-Methylation of *SOCS1* enhancer constructs

All fragments (3µg), including the empty vector, were incubated (4h 37°C) with SssI DNA methyltransferase (16U; New England Biolabs, Ipswich, MA) in a buffer containing 640µM S-adenosylmethionine (New England Biolabs, Ipswich, MA), followed by heat inactivation at 65°C for 20 minutes following the manufacturer instructions. DNA was purified with the phenol/chloroform/ethanol protocol.

Transfection and Luciferase-based reporter gene assays

Human Embryonic Kidney 293 cells (ATCC, Wesel) were cultured in DMEM supplemented with 10% FBS and 2mM of glutamine without antibiotics. Cells were maintained at 37°C in a humidified atmosphere of 5% CO_2.

Lipofectamine LTX was used according to the manufacturer's protocol. 1.25 x 10^5 HEK 293 cells/well were plated in 24 well plates 24 hours before transfection. For transfection, medium was removed and replaced by 100ul of Opti-MEM reduced serum media (Life Technologies) without antibiotics containing the plasmids and 0.75µl of lipofectamine LTX. For each fragment, including empty vector, various concentrations of unmethylated or methylated plasmids were used per well. 10ng, 50ng and 100ng of the test plasmid were co-transfected with 1ng of firefly PG13 promoter plasmid to enable normalization for transfection control. After 24 hour incubation, cells were lysed and luminescence was measured using the Dual Luciferase Reporter Assay System (Promega, Madison) and the GloMax Multi+ Luminometer (Promega, Madison), according to the manufacturer's protocol. The luciferase luminescence was normalized to plasmid input.

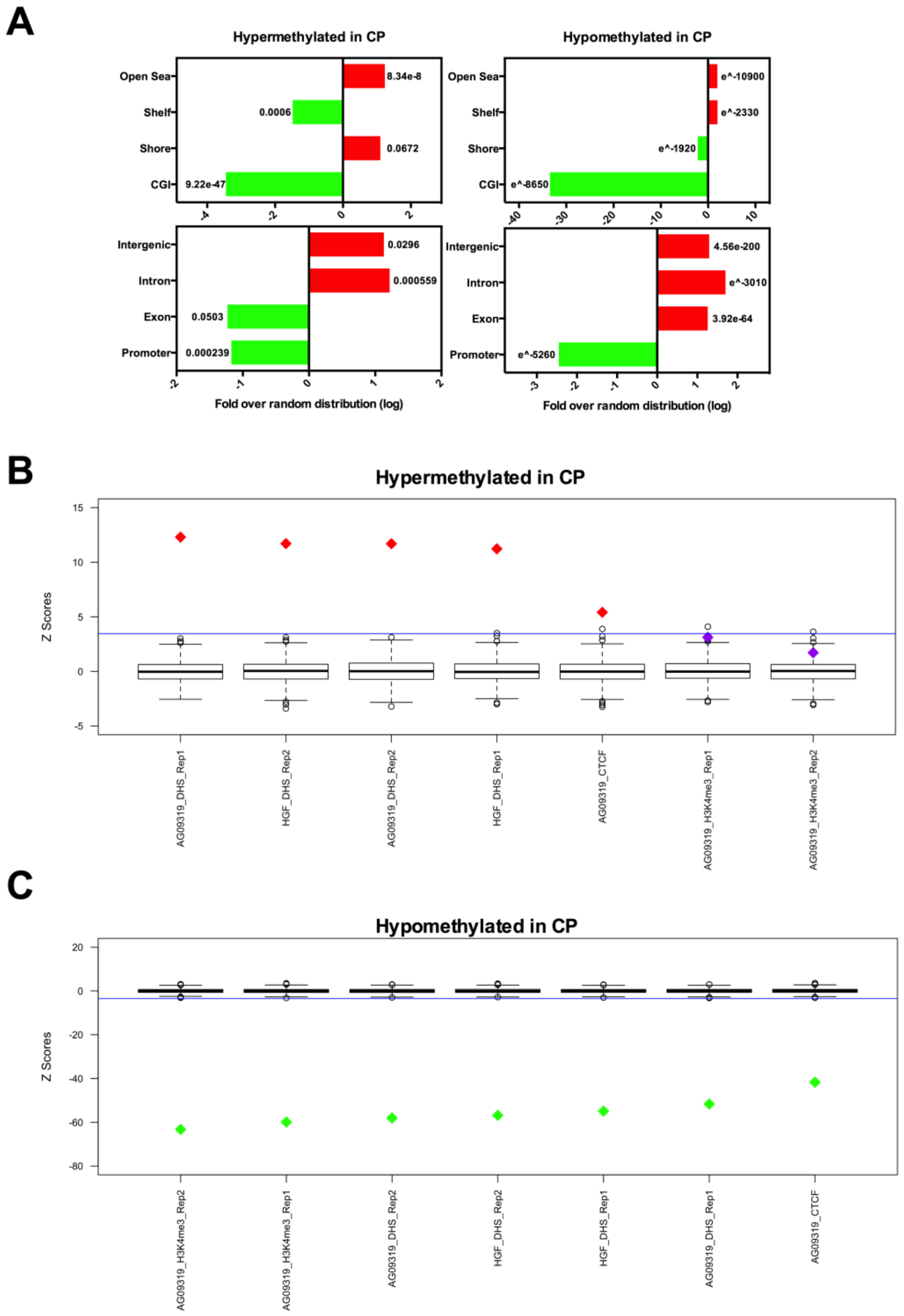

Figure S1 – A) Enrichment of genomics features within hypermethylated and hypomethylated CpG sites. Enrichment was calculated based on the expected distribution of each genomic feature within the array itself. P-values were calculated based on a 1,000 times random permutation analysis. Shores were defined as up to 2kb outside CGIs. Shelfs were defined as up to 2kb outside Shores. **B-C)** Overlap between differentially methylated CpG sites and enhancer (DHS and CTCF) or promoter (H3K4me3) marks in healthy gingival fibroblasts (AG09319 and HGF-1)

from ENCODE. Overlap was computed between each differentially methylated CpG site and the peak for each mark defined by ENCODE. Box-plots represent 1,000 random permutations across the array of the same number of hypermethylated probes (**B**) or hypomethylated probes (**C**). Red diamonds represent the Z-scores of significantly enriched marks and green diamonds represent Z-scores of significantly depleted marks.

A

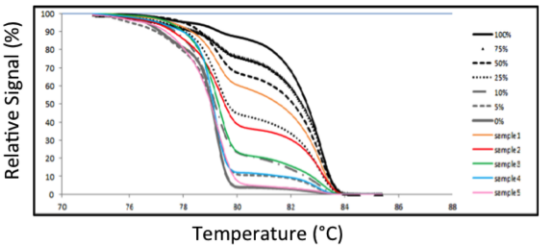

Relative Signal (%)
Temperature (°C)

B

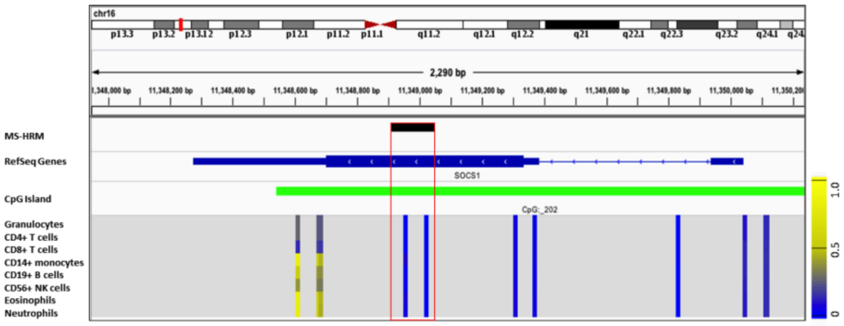

chr16
2,290 bp
MS-HRM
RefSeq Genes
SOCS1
CpG Island
Granulocytes
CD4+ T cells
CD8+ T cells
CD14+ monocytes
CD19+ B cells
CD56+ NK cells
Eosinophils
Neutrophils

C

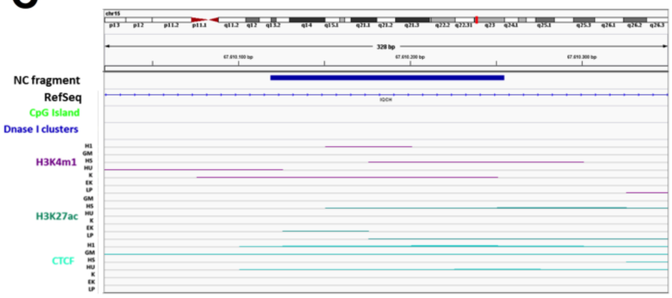

NC fragment
RefSeq
CpG Island
Dnase I clusters
H3K4m1
H3K27ac
CTCF

D

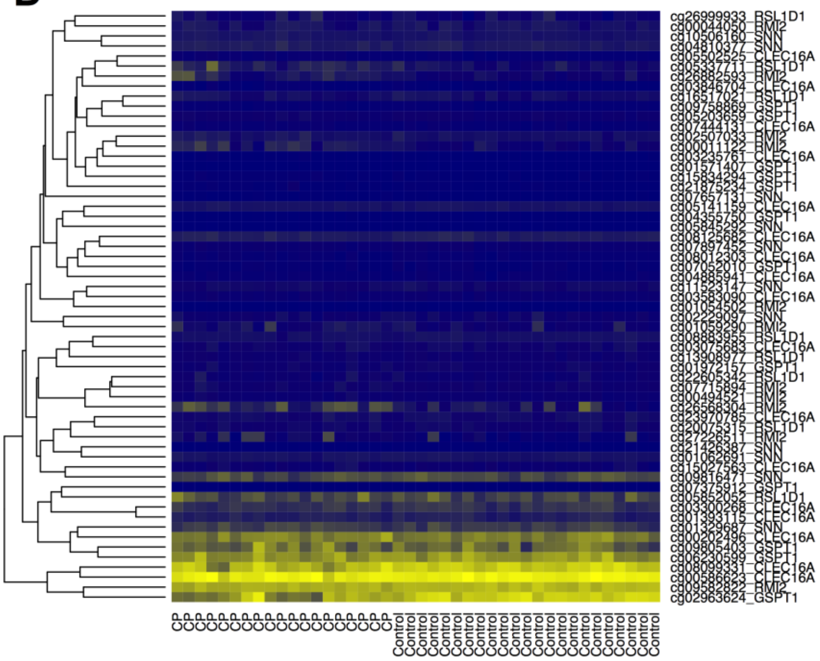

cg26999933_RSL1D1
cg02963624_GSPT1
CP
Control

Figure S2 – A) Melt curves for 5 samples and 7 controls using MS-HRM. **B)** The region on *SOCS1* CGI investigated in MS-HRM is highlighted in red. Public available DNA Methylation (Infinium array 450K) of peripheral blood cells from healthy donors data showed no methylation in the studied region in any immune-inflammatory cell (average). The data is available under GEO 35069. Intensity values were converted to beta-values: 0 (blue) is unmethylated, 1 (yellow) is methylated. **C)** IGV snapshot of the genomic region containing Negative Control (NC) fragment used in the luciferase assay. Note the absence of enhancer markers in all cells analyzed. H1-hESC (H1), GM128878 (GM), HSMM (HS), Huvec (HU) K562 (K), NHEK (EK), NHLF (LF). **D)** Heat-map showing the absolute methylation levels for all the probes located within the promoter region (+/-2.5kb of TSS) of SOCS1 putative target genes (RSL1D1, GSTP1, SNN, CLEC16A and RMI2) in 19 CP tissues and 23 healthy controls. Blue represents unmethylated probes and yellow represents methylated probes. None of these probes showed significant change in DNA methylation between healthy control and CP groups.

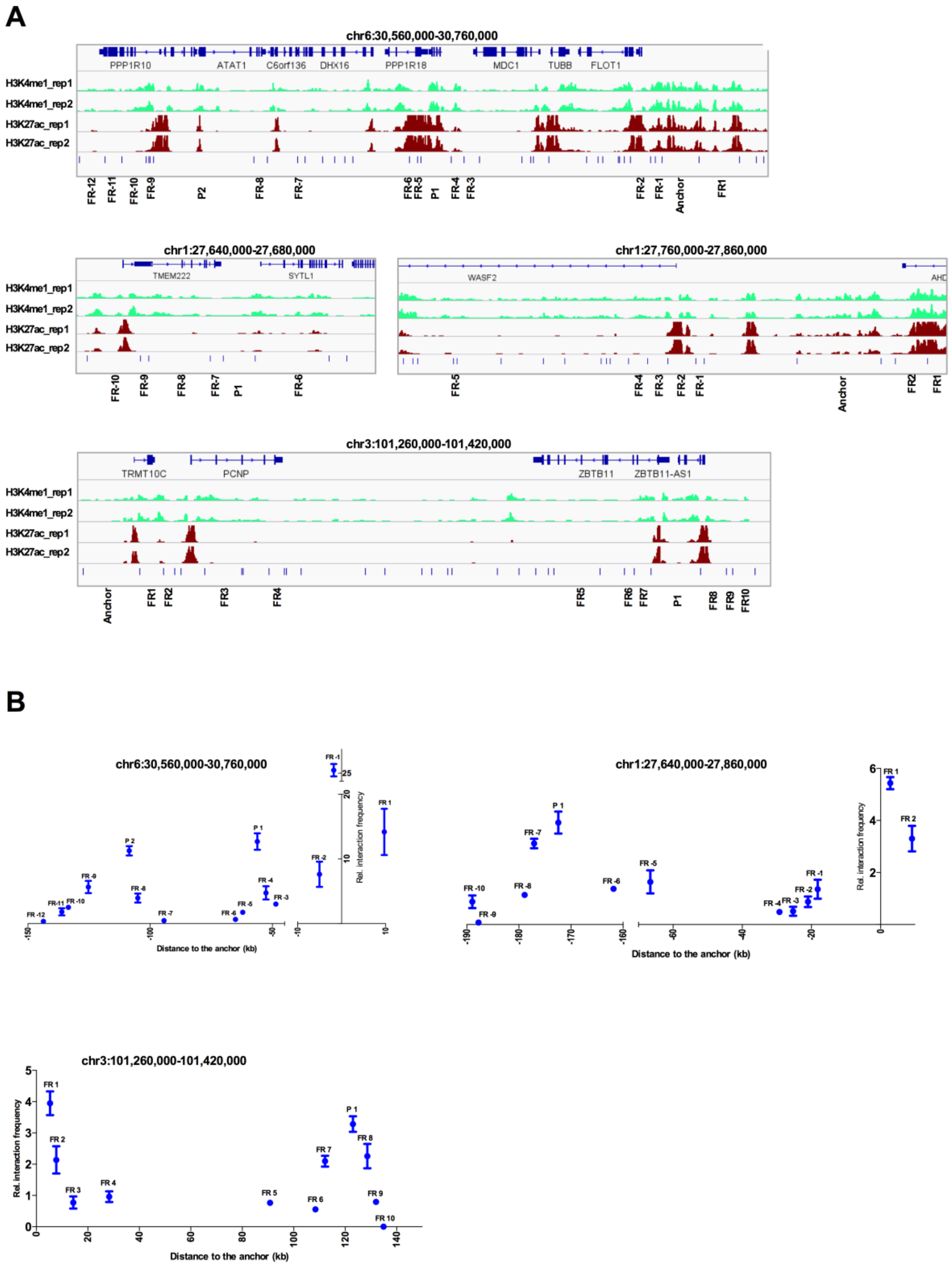

Figure S3 – A) ChIP-seq profile for enhancer-associated marks (H3K27ac and H3K4me1) in normal gingival fibroblasts (AG09319) performed in duplicate for three additional enhancer regions . (Each of these enhancers was hypermethylated in CP). and schematic representation of the 3C assays. Each predicted enhancer element was used as an anchor. Each predicted target promoter was represented as 'P'.

Several Flanking Regions (FR) between the promoters and enhancers were used. **B)** 3C qPCR of long distance interactions assay on Gingival Fibroblasts (AG09319) using SYBR green. The relative interaction frequency of each ligation product to the anchor region has been plotted. Three independent 3C-qPCR experiments were performed. Error bars represent standard error of the mean.

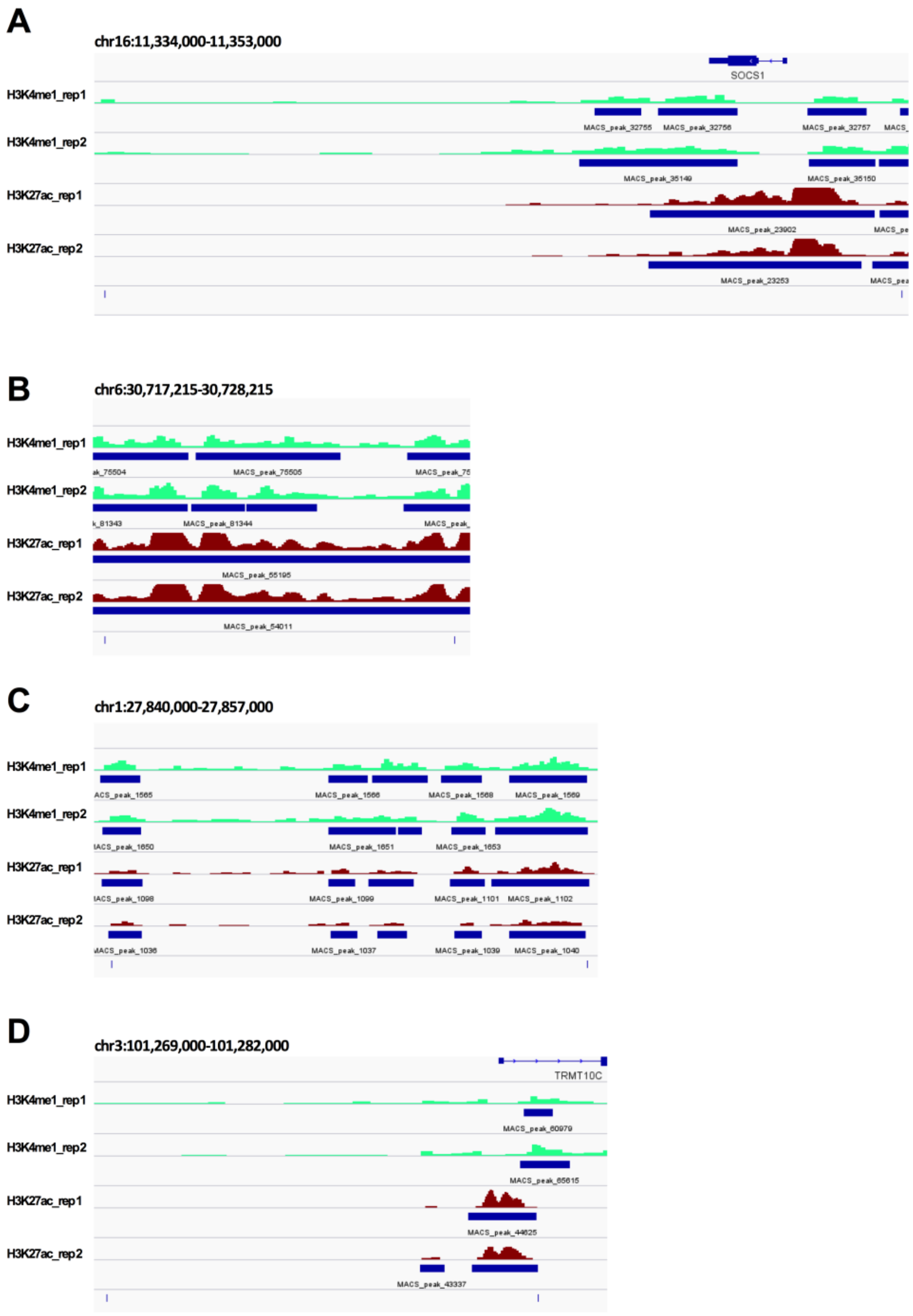

Figure S4 – Higher resolution ChIP-seq profile for enhancer-associated marks (H3K27ac and H3K4me1) for the predicted enhancer located at **(A)** SOCS1 (Figure 6) and the predicted enhancers located at **(B)** chr6:30,717,215-30,728,215 (Figure S3); **(C)** chr1:27,840,000-27,857,000 (Figure S3); and **(D)** chr3:101,269,000-101,282,000 (Figure S3). MACS called peaks are shown as a blue line underneath each track.

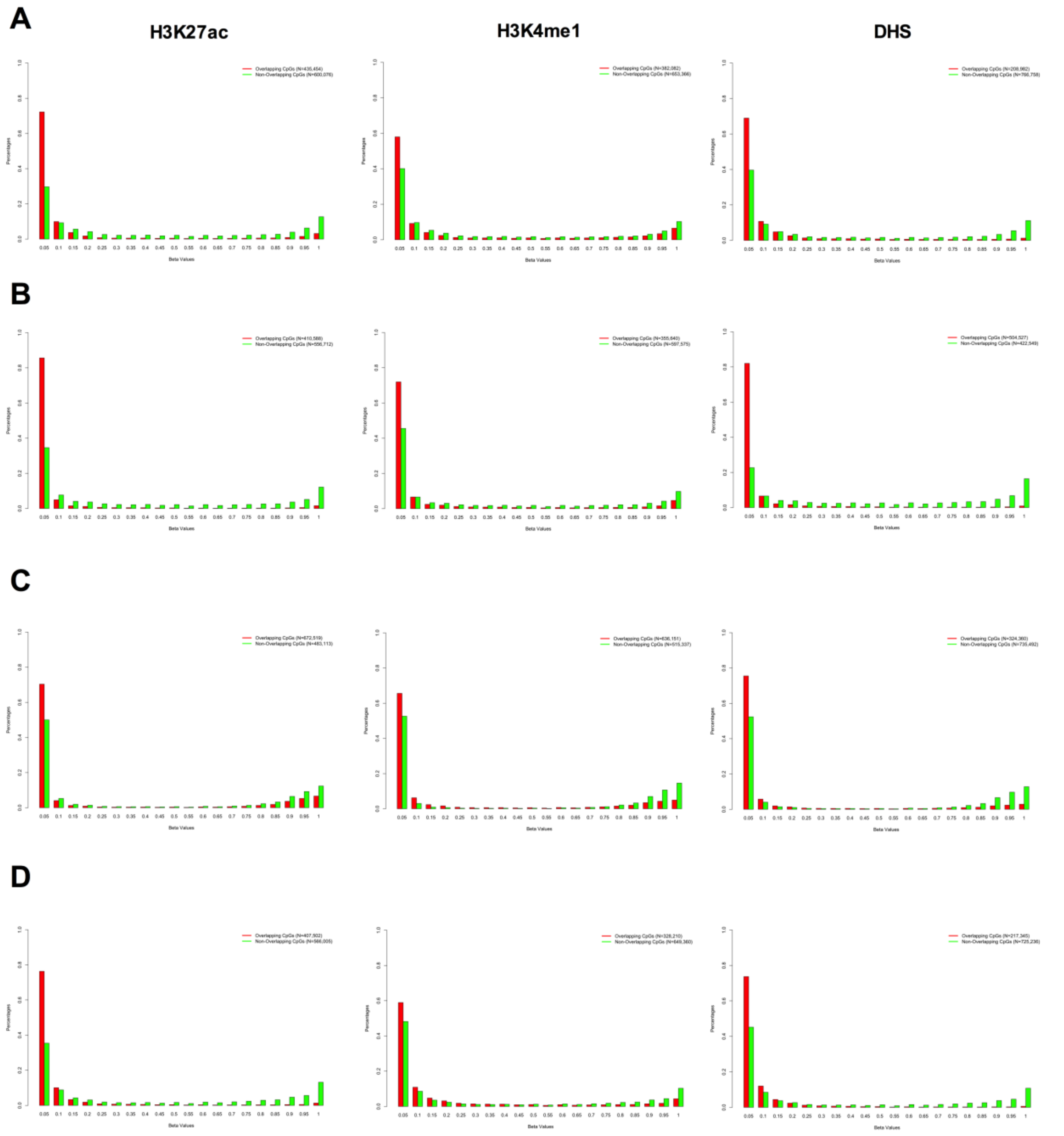

Figure S5 – Enhancer Marks preferentially associate with hypomethylated CpGs. DNA methylation profile of CpGs sites within H3K27ac (left), H3K4me1 (middle) and DHS (right) peaks (red) and DNA methylation profile of CpGs sites outside H3K27ac (left), H3K4me1 (middle) and DHS (right) peaks (green) in K562 **(A)**, GM12878 **(B)**, H1-hESC **(C)** and HSMM **(D)** cells. The DNA methylation profile was obtained by RRBS (Reduced Representation bisulfite Sequencing) from ENCODE database and the enhancer-associated histone marks were obtained by ChIP-seq or DNAse-seq from the same database using ENCODE called peaks. We evaluated the methylation sites of 975,740 CpGs in K562, 927,076 CpGs in GM12878, 1,059,852 CpGs in H1 ESC, and 973,507 CpGs in HSMM with at least

5X coverage on the RRBS assay. There was a significant shift in the DNA methylation profile of overlapping versus non-overlapping CpGs in all twelve panels (Kolmogorov-Smirnov test of Frequency distribution data, p-value$<1.326^{-6}$).

Table S1- Population statistics of CP and healthy individuals

	Healthy	CP
Male/ Fem (%)	36.3/63.3	45.6/54.3
Age±SD	42.54± 11.94	47.17± 11.31

SD: standard deviation, Fem: female, CP: chronic periodontitis

Table S4- Primer sequence used in MS-HRM

Gene	Primer Sequence (5' to 3')	Chromosome position	Amplicon size
SOCS1	F TCGCGGTTGTTATTTAGGTGAAAG<br>R CGAACCCGTAAACACCTTCCTA	Chr 16<br>11348911-11349051	140 bp

F: forward primer, R: reverse primer, Chr: chromosome, bp: base pair, primer sequences are 5' to 3'

Table S5 – Primer sequence for RNA expression

Gene	Primer sequence (5’ to 3’)	Annealing temperature (°C)	Amplicon size
GAPDH	F CCACTCCTCCACCTTTGAC	58	103 bp
	R ACCCTGTTGCTGTAGCCA		
SOCS1	F CTGGGATGCCGTGTTATTTTG	58	224 bp
	R TAGGAGGTGCGAGTTCAGGTC		
RSL1D1	F CGTATTGGTCACGTTGGAATGC	60	93 bp
	R CCACTTCTCTGGCAATTTTTCTG		
SNN	F CTGCTGGTGCAGTATTCGG	60	68 bp
	R CCGTTGGGAGTCATCAGCTTG		
CLEC16A	F ATGCTGCACTACATCCGAGAT	60	86 bp
	F TCGAGTTCGATCACATGGCTC		
GSTP1	F TGGACGCACATTTGATGCC	60	104 bp
	R CCACCTCCTCAATACAGGTATGA		

bp: base pair

Table S6- Primer sequences of *SOCS1* fragments used in the reporter assay.

Fragment	Primer sequence 5'- 3'	Annealing temperature (°C)
**A**	F **CCTAGGATGCATAGT**CGAGGCCATCTTCACGCTA	60
	R **GTTACATGTTGGATC**TGGACGCCTGCGGATTCTA	
**B**	F **CCTAGGATGCATAGT**ACGTAGTGCTCCAGCAGCT	60
	R **GTTACATGTTGGATC**TTCGCCCTTAGCGTGAAGATG	
**C**	F **CCTAGGATGCATAGT**GCCTCGTCTCCAGCCGAG	60
	R **GTTACATGTTGGATC**AGCATTAACTGGGATGCCGTGT	
**AB**	F **CCTAGGATGCATAGT**ACGTAGTGCTCCAGCAGCT	60
	R **GTTACATGTTGGATC**TGGACGCCTGCGGATTCTA	
**NC**	F **CCTAGGATGCATAGT**AGAGGAAGGATTCTGTAGAGAAGTG	60
	R **GTTACATGTTGGATC**CCTAAGGGAAGCCGTGTGTAG	

In bold, 15 base pair sequence complementary to the ends of linearized vector (Infusion kit)

Table S7- Primer sequences used in 3C-experiments

3C Fragments	Primer sequences F - R	
Chr 16		
Anchor/FR 1	GGTCTCGAACTCCTGTCCTTTAG	CTGGTGGCAGTGAGGAGAAC
FR 2	GGCACCATTAGCCTCCCATT	CTGCAGGGAAGGGAAATGGAC
FR 3	TCAGCACTGGTTCCCTGATTTG (f)	CACGCGCAGTTCACAATGAG
FR 4	TCAGCACTGGTTCCCTGATTTG	CACGCGCAGTTCACAATGAG
P 1	CAAGCATGGCACCAGTATCTGT	AGTGGGAGGTGTCTGGGTTATG
FR 5	AGCCGGGCAGATTGGAAAGTAT (f)	GCTCATAGTCACACGGCAAGGT
FR6	AGCCGGGCAGATTGGAAAGTAT	GCTCATAGTCACACGGCAAGGT
FR 7	AGAGACATTCAAGGGAGAAAGGAAC	GCAGTAGTGATAGGTTTTAGAGACG
FR 8	CCACAATCACCCCAGTTGAGAAG	GCCAGCCCCACTTGATATCCAT
FR 9	GATGGGATCTCTCTGTGTTGCC	CCACTCTCACAGGCCTATGC
FR 10	AAAGCATAACCCCAGATCCTCCT	TCAGGTATCCCAGACATGCACA
P 2	CTAGCTCAGCAGTACCGTGTCT	CCTCTGTGCATCCTTCCAGAGA
FR 11	TGAGGCAGGAAAATGGCGTGAA	TGCTGCCGAGATTTGCTTGTAG
FR 12	AGCCTCACCACACTCTCTACAC	GCCACCACGCTCAGCTAATTT
FR 13	AGCCAGAGTGACAGGACAAGA	GGTAAGAAAGTCCCCGTTGTGA
FR 14	ACACACAGGCATTCAGAACACA	CCGCCTTCTGAGCAGTTTTGAT
FR 15	GATACCATTTCGCCTGAATTAAATCCT	ATGCTTTCCTCGGTTTCTTTTCAC
FR 16	TATCCCATCCCCACATATATTAAC	CCCTGTCTCAGGCCATTCTAATG
FR 17	TGGGCACTTTTAGGAGCTGAGA	AGCACGGTTGATTTGGTGGTTT
FR 18	CTGATGCACGAACACAGTGAGT	TTACCCTTAGCATTGTGTACGCTT
FR -1	ACGGGTATGAGCCAGCACCTAT	TGCCAGCAAAGCCAACATCAGA
FR -2	TGGGTGACAGAGAGACTCCATTT	AGTGTGAAAGAGCCAAGACCAGTT
FR -3	TGGGTGACAGAGAGACTCCATTT	AGTGTGAAAGAGCCAAGACCAGTT (r)
FR -4	CGCCAGCCTCTGTGATGTTCAG	GGCCTCCCAAAGTGCTAGGATT
FR -5	AGGTGATGAGCAGGGTCCAT	CAGCCACCCAAGTTTCTGTGA
FR -6	AGCCCTGCCTATACCCTACAC	TGCCCTGAAAACCACCATCTG
P 3	CATCTACGCCGATTCCCTCTC	CGCTGGGAAATGCAGTTCAC
FR -7	TCGGGGTCTCCTTCCTTTTCAC	AGCAAGGGAGAGGGAGGAATTC
FR -8	TGGCACTTGGTTCTCGCTAAGA	CACCCCGCAATACCATCATTTG
Chr 1		
FR 2	CTCCTGCCTCCTGTTACCCTTT	GCTGACGGGGAAGACAACACTA
FR 1	ACCGTTGGACCTGGAATTCACT	GCGGTGACCCTCGATCATGAC
Anchor	TTAGCAAGTGACAGGGAGGTTGAC	ACCATCAGCCCATTTTCCATTTGG
FR -1	CCTCCCCAGTTCAAGCAATTCTC	ATCACTGGAGGTCTGGAGTTCAC
FR -2	TTCTCGTAGTCCCTGAAAGAATGC	TGAGACACAGTGAGGTTTGATGAC
FR -3	CCCATAGCCGTCCCTACTATTCTG	CCATCAGAGGGCCACATTTTCTTG
FR -4	ACCAGTCCACCTCACTCTACAGT	CCTCGGCCTCCCAAAGTTCTG
FR -5	ATTTTCCCTCAGTGGCCTTCTG	GCTCTTGCCATTTCTGAATTTTGG
FR -6	TACATTGGCTTCAGGACCATGAAC	GGTCTATGAGGAGCCCACTAATCT (r)
P 1	TACATTGGCTTCAGGACCATGAAC	GGTCTATGAGGAGCCCACTAATCT
FR -7	TTGGTGCCCTGTCATTGTTTTCTC	AAGCCTACAAGTCCTTCCCAACAG

FR -8	ACTCGTGAGCCAGCATAGGTTAG	CAACCTCTGTGTGACCAGTTATTCTC
FR -9	ACCTCATGCTAGCCTCACGAAA	CTGGCCATGCAGACTCACCAA
FR -10	TGCAGGCTATACAGGAAGCATAGT	GGTGGGATCATGAGGCTGGTTT
Chr 3		
Anchor/FR1	ACTTGAAGTGTGAGGTGAAGAATAGC	AAACACCTCAAACGCAACAAATCG
FR 2	TTCCGTGCCCAAATTCCCTCTT	TGTGTAATTTGTGAGGCGACAACT
FR 3	CCGGCCAACATTAATTTCTGTCTAT	GAGGTGTGCAGCCTAAATGAGAAG
FR 4	TTCTCCATTGTTGCTGTTGTCATG	CTGGGACTGACTCTACTTGAATCAC
FR 5	TTGAACTCCTGGCCTCAAGTGATC	GTGACAACTAAGCATGACCCTGAAC
FR 6	AGAAGAACGTTTTACCACTCCTTTG	TTAGTAAGAAGAGGCAGCCAGAAG
FR 7	TCC ACT TAC CGA CTG TCC CTT T	CCT TGT GGG CCT CAG TTT TGT A
P 1	AGAGGCTGAAAAGGGAGGATCATC	TTGTTTGGATGGGTTTGGGTAGAC
FR 8	CAATTGAAGGAGGCTGAGCAGATG	AGAATCCTTCCCACACCTGAGAAG
FR 9	AGGCTTTTCTCTAGGGTGAAGGT	TGCTTGTTTTAGTTCCTCGCCAAT
FR 10	GATGGCACGCATGTTCCTTCAT	AGTTGCATCTGAGGAGTGGTACA
Chr 6		
FR 1	GAGAGCAAGCATTCTAGGCATTCG	ATTGAGAGCCTGGGAAGTGTCTAG
Anchor	CTGAGAAAGGAAAGCAGGGTGAAG	AGCTGGCACTTAAACCCAATTTCG
FR -1	TCCACTGGAGGCTGTGTCATAC	CCTAAGTGGGAGGGGAGAAAAGTG
FR -2	TGATCTCGACTCACTGCAACCT	CCATCTCAGGACACCACTACACT
FR -3	AGCCAAGACTGCTCCACTGTAC	GAAGCTGGGTCACTTGTCCTTTAG
FR -4	TGC AAC ACC TTT CGG ACC TTT G	GCT GAA GAT CAA AGG CCC GAG AA
P 1	GGAGAAACCCTGTCTCTACCAA	GTG GCA GAC AGT ATG ACC CAG AT
FR -5	CAGCTCTTCACTCCGTTGTTGTTG	GAGCCTCTCCTTCCTGTTCTCTGT
FR -6	CTCCTTCTCAGCCTCCCCTTCT	CCCAGGGACATAGAGGCTCAAAC
FR -7	CCC TGC TCT CTG GCC TAG GTA T	CTC AGG GCT GGG CAG ATG AAA A
FR -8	CTT GGG CAT GTG GTA GCG ATT G	TCC CTG AGC TGC GGA AGA AAT C
P 2	GCA CCT CAA CTC GCT TTG GTT T	CTC CCG GCT TTC CCT CTC AAG
FR -9	CTG TGT GTG GGC TTC CGT ATC T	AGT GGA GCA GAC GTC AGG TAA T
FR -10	AGCCCTTGTGTCTTTCCCATCT	GGCTGTCCATCTCTGTGTAGTTCT
FR -11	GCC CCT ACC CCT CAG TCA TTT T	GGA GAT GGG AAG CCA GGT CTA C
FR -12	GTGTGGGCAGCTGAACTCAAAC	AGTCATGCCAAGTTCCGTTCCA

Supplementary References

1. Armitage GC. Periodontal diagnoses and classification of periodontal diseases. Periodontology 2000. 2004; 34:9-21.
2. Armitage GC. Development of a classification system for periodontal diseases and conditions. Annals of periodontology / the American Academy of Periodontology. 1999; 4(1):1-6.
3. Wojdacz TK, Dobrovic A and Hansen LL. Methylation-sensitive high-resolution melting. Nature protocols. 2008; 3(12):1903-1908.
4. Thurman RE, Rynes E, Humbert R, Vierstra J, Maurano MT, Haugen E, Sheffield NC, Stergachis AB, Wang H, Vernot B, Garg K, John S, Sandstrom R, Bates D, Boatman L, Canfield TK, et al. The accessible chromatin landscape of the human genome. Nature. 2012; 489(7414):75-82.
5. Demmer RT, Behle JH, Wolf DL, Handfield M, Kebschull M, Celenti R, Pavlidis P and Papapanou PN. Transcriptomes in healthy and diseased gingival tissues. Journal of periodontology. 2008; 79(11):2112-2124.
6. Aryee MJ, Jaffe AE, Corrada-Bravo H, Ladd-Acosta C, Feinberg AP, Hansen KD and Irizarry RA. Minfi: a flexible and comprehensive Bioconductor package for the analysis of Infinium DNA methylation microarrays. Bioinformatics. 2014; 30(10):1363-1369.
7. Johnson WE, Li C and Rabinovic A. Adjusting batch effects in microarray expression data using empirical Bayes methods. Biostatistics. 2007; 8(1):118-127.
8. Hagege H, Klous P, Braem C, Splinter E, Dekker J, Cathala G, de Laat W and Forne T. Quantitative analysis of chromosome conformation capture assays (3C-qPCR). Nature protocols. 2007; 2(7):1722-1733.
9. Zhang X, Cowper-Sal Lari R, Bailey SD, Moore JH and Lupien M. Integrative functional genomics identifies an enhancer looping to the SOX9 gene disrupted by the 17q24.3 prostate cancer risk locus. Genome research. 2012; 22(8):1437-1446.
10. Court F, Baniol M, Hagege H, Petit JS, Lelay-Taha MN, Carbonell F, Weber M, Cathala G and Forne T. Long-range chromatin interactions at the mouse Igf2/H19 locus reveal a novel paternally expressed long non-coding RNA. Nucleic acids research. 2011; 39(14):5893-5906.
